# Development of plant extracts as substrates for untargeted transporter substrate identification in *Xenopus* oocytes

**DOI:** 10.3389/fpls.2025.1640426

**Published:** 2025-09-17

**Authors:** Christos Theodorou, Victor de-Prado-Parralejo, Deyang Xu, Yoshimasa Todoroki, Louise Svenningsen, Tetsuya Mori, Christoph Crocoll, Masami Yokota Hirai, Hiroshi Tsugawa, Hussam Hassan Nour- Eldin, Barbara Ann Halkier

**Affiliations:** ^1^ DynaMo Center, Department of Plant and Environmental Sciences, University of Copenhagen, Frederiksberg C, Denmark; ^2^ Department of Biotechnology and Life Science, Tokyo University of Agriculture and Technology, Koganei-shi, Tokyo, Japan; ^3^ RIKEN Center for Sustainable Resource Science, Yokohama, Kanagawa, Japan; ^4^ RIKEN Center for Integrative Medical Sciences, Yokohama, Kanagawa, Japan

**Keywords:** non-targeted metabolomics, mass spectrometry, transportomics, oocytes, transporters, plant extracts

## Abstract

Amongst the thousands of transport proteins constituting approximately 10% of coding sequences in a genome, only few have an assigned function. Heterologous expression of transporters in combination with the use of plant extracts as complex mixtures of substrates, can be a powerful tool for untargeted identification of plant transporter functionality. In this study, we developed and evaluated four extraction protocols to generate *Arabidopsis thaliana* seedling extracts for use as substrate mixtures in high-throughput screening of transporters expressed in *Xenopus laevis* oocytes, a well-established system for transporter studies. To expand chemical space of plant extracts for transporter assay, we prepared Arabidopsis seedlings extracts from liquid culture-grown plants subjected to biotic stress (flagellin 22 and chitin treatments to mimic bacterial and fungal infections) and abiotic stress (phosphorus and nitrogen starvation). Extracts from these treatments were characterized and subsequently combined into a single metabolic mixture to capture treatment-specific metabolites and expand the metabolic space. Toxicity testing of the pooled extract against *X. laevis* oocytes revealed that a liquid-liquid extraction protocol, facilitating lipophilic compound removal, outperformed single solvent extractions in terms of metabolite repeatability and reduced membrane permeation. Metabolite profiling of the final extract using pure standards, structural databases, and in-silico tools identified over 200 metabolites. Our study highlights the importance of developing metabolically diverse yet low-toxicity plant extracts as a critical step toward advancing plant transporter substrate screening. The optimized extraction protocol in combination with *X. laevis* oocyte assays, provide a robust platform for the functional characterization of plant transporters, paving the way for deeper insights into plant physiology and metabolism.

## Highlights

- Development of a two-phase liquid-liquid extraction method to generate metabolically diverse *Arabidopsis thaliana* seedling extracts.
- Optimized extracts for high-throughput functional screening of plant transporters using *Xenopus laevis* oocytes.
- Identified a protocol that minimizes oocyte membrane permeation while maximizing metabolite coverage and repeatability
- Combined extracts from biotic (flagellin 22, chitin) and abiotic (phosphorus, nitrogen starvation) stress treatments to expand metabolite diversity.
- Identified more than 200 primary and secondary metabolites based on a combinatorial technique

## 1 Introduction

The ability to move metabolites across membranes is essential to all life to maintain cellular homeostasis and to enable signaling. However, amongst the thousands of transport proteins that constitute approximately 10% of coding sequences in the contemporary genomes (Saier Jr. and Ren, 2006), most remain with no assigned function. To fill this knowledge gap there is a need for effective experimental approaches for assignment of substrates to transporters.

Over the past two decades, heterologous expression of transporters in model organisms, exposure to metabolite extracts as complex substrate mixtures, and detection of transport activity via untargeted liquid chromatography-mass spectroscopy has emerged as a so-called transportomics tool for untargeted identification of human (Krumpochova et al., 2012) and plant transporters’ substrates (Demurtas et al., 2020). In plants, vacuolar transporters of crocin in *Crocus sativus* were identified by expressing a selection of target transporters in yeast microsomes and exposing them to extracts from crocus (Demurtas et al., 2019). Additionally, we lately used oocytes from *Xenopus laevis* (hereafter *Xenopus)* as expression hosts in combination with metabolite extracts from seeds from the model plant *Arabidopsis thaliana* (hereafter Arabidopsis) to identify novel substrates for members of the nitrate and peptide transporter family (NPF) (de Prado Parralejo, 2025). As shown for mammalian transporters, these studies demonstrated that the use of metabolite extracts allows for unbiased screening for transport activity towards hundreds of endogenous metabolites, thereby alleviating the limitations posed by the commercial unavailability of most plant metabolites. Due to the ease by which transporters are expressed in *Xenopus* oocytes (Larsen et al., 2017) this prompted us to embark on determining the substrate specificity of the entire transportome of *Arabidopsis* by using metabolite extracts as substrate mixtures and *Xenopus* oocytes as expression hosts.

As a first step, we present here a robust methodology for the generation and characterization of plant extracts as substrate mixtures for plant transporter assays in *Xenopus* oocytes. We examined four different protocols for extracting metabolites from Arabidopsis seedlings and compared the amount and reproducibility of the metabolites captured. The protocols were evaluated based on their metabolic content (number of metabolic features captured by each protocol) inter-and intraday precision, and toxicity towards the oocytes for the selection of the best-performing protocol (**Supplementary Figure 1A**). To expand the metabolic diversity, we used the selected protocol to generate extracts from seedlings with four different stress mimicking-treatments. The extracts were combined and compared to the untreated extract to evaluate if the inclusion of the different treatments increased the final metabolic diversity captured by our extraction. In addition, we performed an in-depth metabolite annotation with the combination of pure standards, spectral libraries, Molecular Networks and in-silico tools. (Tsugawa et al., 2019 to achieve as many high-confidence metabolite identities as possible (**Supplementary Figure 1B**, **Supplementary Table 1**).

## 2 Materials and methods

### 2.1 Plant material sterilization and growth

Seeds from wild-type Arabidopsis thaliana ecotype Colombia 0 (Col-0) were used for plant growing under no treatment and with biotic and abiotic stresses. 20 mg of seeds were sterilized with 1mL 70% ethanol containing 0.01% Triton X-100 for 10 minutes. After incubation the solution was removed, and seeds were washed with 96% EtOH and left to dry in sterile conditions. 1 mL of sterile Milli-Q water was added to the tubes containing the seeds and left for stratification at 4°C for 4 days.

A. thaliana wild-type Col-0 seeds (20 mg) were grown in 200 ml of sterile liquid Murashige-Skoog (MS) media in a 1-liter Erlenmeyer glass flask on orbital shakers with constant light (120 µE) and temperature (21°C) for a total of 16 days. The shaker speed was kept low (50 rpm) and was gradually increased to 80 rpm after three days, and to 100 rpm after seven days of growth. After 7 days, 50 mL of fresh MS medium was added to the seedlings. On day 14, the media were discarded, and seedlings were washed with 200 mL fresh media (either normal MS media or MS with nutrient deficiency). The macronutrient deprivation and biotic treatments took place as described previously (Scheible et al., 2004; Bielecka et al., 2015) with some modifications, and lasted for 2 days. In short:

- For the untreated plants, 300 ml of fresh MS media were added. The MS media composition was the following: NH_4_NO_3_; 1650 mg/l, H_3_BO_3_; 6.2 mg/l, CaCl_2_; 332.2 mg/l, CoCl_2_*6H_2_O; 0.025 mg/l, CuSO_4_*5H_2_O; 0.025 mg/l, Na_2_-EDTA; 37.26 mg/l, FeSO_4_*7H_2_O; 27.8 mg/l, MgSO_4_; 180.7 mg/l, MnSO_4_*H_2_O; 16.9 mg/l, Na_2_MoO_4_*2H_2_O; 0.25 mg/l, KI; 0.83 mg/l, ZnSO_4_*7H_2_O; 8.6 mg/l, KNO_3_; 1900 mg/l, KH_2_PO_4_; 170 mg/l.
- For the inorganic Nitrogen starvation, normal MS media were replaced by 300 ml inorganic Nitrogen (- N) deficient media with the following composition: H_3_BO_3_; 6.2 mg/l, CaCl_2_; 332.2 mg/l, CoCl_2_*6H_2_O; 0.025 mg/l, CuSO_4_*5H_2_O; 0.025 mg/l, Na_2_-EDTA; 37.26 mg/l, FeSO_4_*7H_2_O; 27.8 mg/l, MgSO_4_; 180.7 mg/l, MnSO_4_*H_2_O; 16.9 mg/l, Na_2_MoO_4_*2H_2_O; 0.25 mg/l, KI; 0.83 mg/l, ZnSO_4_*7H_2_O; 8.6 mg/l, KNO_3_; KH_2_PO_4_; 170 mg/l, K_2_SO_4_; 3278 mg/l.
- For the inorganic Phosphorus starvation, normal MS media were replaced by 300 ml inorganic Phosphorus (- P) deficient media with the following composition: NH_4_NO_3_; 1650 mg/l, H_3_BO_3_; 6.2 mg/l, CaCl_2_; 332.2 mg/l, CoCl_2_*6H_2_O; 0.025 mg/l, CuSO_4_*5H_2_O; 0.025 mg/l, Na_2_-EDTA; 37.26 mg/l, FeSO_4_*7H_2_O; 27.8 mg/l, MgSO_4_; 180.7 mg/l, MnSO_4_*H_2_O; 16.9 mg/l, Na_2_MoO_4_*2H_2_O; 0.25 mg/l, KI; 0.83 mg/l, ZnSO_4_*7H_2_O; 8.6 mg/l, KNO_3_; 1900 mg/l.
- For the flagellin-related experiments; normal MS media (recipe above) were replaced with 300 ml MS media spiked flagellin-22 in a final concentration of 1 µM flg22. The flagellin-containing media were generated by mixing flagellin 22 (Genscript) with MilliQ water.
- For the chitin-related experiments; normal MS media were replaced with 300 ml MS media containing chitin in a final concentration of 0,1 mg/ml. The chitin solution was generated by dissolving chitin (Merck, Darmstadt, Germany) in MilliQ water at 10 mg/ml with gentle shaking together with metal beads for a few hours at 4 °C. The resuspended chitin solution was autoclaved at 121°C for 20 min. The solution was centrifuged (3000 rpm at 2 min) to obtain a clear supernatant, which was added to the flask to a concentration of 0,1 mg/mL.

After induction of the different treatments, plants were allowed to grow for two days until day 16, when they were harvested. Excess media were removed, and seedlings were washed three consecutive times in an excess of deionized water for a duration of one minute/wash. After the wash, seedlings were blotted on tissue paper gently and were immediately snap frozen into liquid nitrogen. The snap-frozen seedlings were stored at -80°C until freeze-drying.

### 2.2 Plant material preparation and experimental design

#### 2.2.1 Freeze drying and pulverization

The frozen seedlings were freeze-dried on a LyoCube benchtop freeze-dryer (CHRIST-Gamma LSC plus) for 48 hours (24 hours pre-drying at -25°C followed by 24 hours main drying at 20°C) until all water was removed. After completion, the freeze-dried seedlings were split into 50 ml Eppendorf tubes containing 10- 3mm diameter metal beads (Bearing Warehouse) and were pulverized into fine powder in a commercial SK550 paint-shaker (FAST & FLUID) at maximum operating speed over three cycles of operation lasting 3 minutes each.

#### 2.2.2 Experimental design

For the generation of the plant material, two individual plant batches were grown in parallel under the same conditions. Each plant batch included eight flasks of untreated plants and eight flasks of plants under each treatment, respectively, leading to a total of 40 flasks per plant batch. Plant material from different flasks was considered as biological replicates. The comparison of the different treatments was performed on five randomly selected biological replicates regardless of the plant batch.

Regarding the comparison of the different extraction protocols, half of the freeze-dried plant material, corresponding to the untreated plants from the different plant batches, was pooled together into a homogeneous powder. After pooling, the plant material was equally split into different vials to generate technical replicates that were used for the comparison of the extraction protocols. The intraday precision for every extraction protocol was calculated based on measurements among technical replicates generated as described above (n = 5/extraction protocol). For the interday precision, aliquots of the same five technical replicates were measured on three different days (day 1, day 20, and day 35), resulting in a total of 15 measurements (n-total = 15/extraction protocol).

### 2.3 Metabolite extraction protocols

All solvents used for the four extraction protocols (Acetonitrile, Methanol, Ethanol, Dicloromethane) were bought from Merck (Merck, Darmstadt, Germany). The four different extraction protocols were conducted in parallel. Each extraction protocol was performed in five technical replicates of the untreated plant material.

#### 2.3.1 Extraction protocol A

Freeze-dried plant material (100 mg.) was carefully weighed into different 5 ml Eppendorf tubes kept in dry ice and pre-filled with two 3mm diameter metal beads (Bearing Wearhouse). 1.5 ml of Acetonitrile/Methanol (1:1) spiked with 3 μM of internal standard Ampicillin (Merck, Darmstadt, Germany) were added to the tube. The samples were vortexed for 30 seconds and subsequently extracted in a water bath sonicator (Vevor, Shanghai, China) at room temperature for 20 minutes at 60 Hz. The samples were then centrifuged at 4°C at 5000 rpm for 20 minutes. After centrifugation, the supernatant was removed into a 12-ml DURAN glass tube (Hounisen, Skanderborg, Denmark), and the plant material was re-extracted with 1 ml of Acetonitrile/Methanol/Water (4:4:2) spiked with 3 μM of Ampicillin using the same steps. After the second extraction, the supernatant was pooled with the previous one, and a final extraction using 1 ml of Methanol/Water (8:2) spiked with internal standard was conducted similarly. The third supernatant was pooled with the previous two, and the samples were filtered through 0.22 μm PVDF filter pre-conditioned with 300 μl of 50% methanol. After filtration, samples were dried overnight under a mild stream of nitrogen.

#### 2.3.2 Extraction protocol B

Freeze-dried plant material (100 mg.) was carefully weighed into different 5 ml Eppendorf tubes kept in dry-ice and pre-filled with two 3mm diameter metal beads. 1.5 ml of Ethanol/Methanol (1:1) spiked with 3 μM of internal standard Ampicillin (Merck, Darmstadt, Germany) were added to the tube. The samples were vortexed for 30 seconds and subsequently extracted in a water bath sonicator (Vevor, Shanghai, China) at room temperature for 20 minutes at 60 Hz. The samples were then centrifuged at 4°C at 5000 rpm for 20 minutes. After centrifugation, the supernatant was removed into a 12-ml DURAN glass tube (Hounisen, Skanderborg, Denmark), and the plant material was re-extracted with 1 ml of Ethanol/Methanol/Water (4:4:2) spiked with 3 μM of Ampicillin using the same steps. After the second extraction, the supernatant was pooled with the previous one, and a final extraction using 1 ml of Methanol/Water (8:2) spiked with internal standard was conducted similarly. The third supernatant was pooled with the previous two and the samples were filtered through 0.22 μm PVDF filter pre-conditioned with 300 μl of 50% methanol. After filtration, samples were dried overnight under a mild stream of nitrogen and stored at -80°C.

#### 2.3.3 Extraction protocol C

Freeze-dried plant material (100 mg.) was carefully weighed into different 5 ml Eppendorf tubes kept in dry-ice and pre-filled with two 3mm diameter metal beads. 1.5 ml of Methanol/Water (8:2) spiked with 3 μM of internal standard were added to the tube. The samples were vortexed for 30 seconds and subsequently extracted in a water bath sonicator (Vevor, Shanghai, China) at room temperature for 20 minutes at 60 Hz. The samples were then centrifuged at 4°C at 5000 rpm for 20 minutes. After centrifugation, the supernatant was removed into a 12-ml DURAN glass tube (Hounisen, Skanderborg, Denmark), and the plant material was re-extracted with 1 ml of the same solvent using the exact same steps. After the second extraction the supernatant was pooled with the previous one and a final re-extraction using 1 ml was conducted similarly. The third supernatant was pooled with the previous two, and the samples were filtered through 0.22 μm PVDF filter pre-conditioned with 300 μl of 50% methanol. After filtration, samples were dried overnight under a mild stream of nitrogen and stored at -80°C.

#### 2.3.4 Extraction protocol D

Freeze-dried plant material (100 mg.) was carefully weighed into different 5 ml Eppendorf tubes kept in dry-ice and pre-filled with two 3mm diameter metal beads. 1.5 ml of Dichloromethane/Methanol (3:1) spiked with 3 μM of internal standard were added to the tube. The samples were vortexed for 30 seconds and subsequently extracted in a water bath sonicator (Vevor, Shanghai, China) at room temperature for 20 minutes at 60 Hz. The samples were then centrifuged at 4°C at 5000 rpm for 20 minutes. After centrifugation, the supernatant was removed into a 12-ml DURAN glass tube (Hounisen, Skanderborg, Denmark), and the plant material was re-extracted with 1 ml of the same solvent using the exact same steps. After the second extraction the supernatant was pooled with the previous one and a final re-extraction using 1 ml of Methanol/Water (8:2) spiked with 3 μM of internal standard was performed. The third supernatant was pooled with the previous two into the 12 ml glass tubes and the samples were filtered through 0.22 μm PVDF filter pre-conditioned with 300 μl of 50% methanol followed by the addition of 1 ml of milliQ water to induce phase separation. The tube was left to stand in room temperature for 5 minutes and was subsequently centrifuged at 3000 g at 4°C for 20 minutes. After centrifugation, the upper MeOH-H_2_O was removed carefully in a new 12-ml glass tube, while the lower DCM (Dichloromethane) phase was re-extracted with 1.5 ml of water with the same procedure as the previous step. After the second phase separation, the upper phase containing the secondary metabolites was pooled with the previous upper phase, and samples were dried overnight under a mild stream of nitrogen and stored at -80°C.

### 2.4 Toxicity assays

Each oocyte (n=10) was injected with nuclease-free water (Ambion) using a Drummond NANOJECT II (Drummond scientific company, Broomall Pennsylvania). Water-injected oocytes were incubated for 1 hour at 16°C in plant extracts resuspended in Kulori buffer pH 5 (90 mM NaCl, 1 mM MgCl_2_, 1 mM CaCl_2_, 1mM KCl, 5 mM 4-Morpholineethanesulfonic acid (MES)) with 5% EtOH supplemented with amikacin (100 ug/mL). After incubation, oocytes were washed three times in Kulori buffer pH 5 and one time with MilliQ water and homogenized in 50% methanol containing 2500 nM *p*-hydroxybenzyl glucosinolate (pOHB) as internal standard for targeted glucosinolate analysis. For oocyte permeation data, the glucosinolate MRM transition that displayed a higher signal relative to the oocyte matrix was chosen to illustrate phytochemical permeation. Normality of the data was assumed, and outliers were excluded from analysis based on the Grubbs method with a significance level of p < 0.05. For the analysis, one-way ANOVA followed by Tukey t-test was used to compare significant differences.

### 2.5 Impact of a/biotic treatments on the metabolome

To assess the impact of the different treatments on the metabolome we performed a comparative metabolic profiling of the conversely treated plants. The selected extraction protocol (Protocol D) was performed in parallel to treated and control freeze-dried plant material. In short, 100 mg of freeze-dried control and treated plant material (n = 5 biological replicates/sample group) was carefully weighted into different 5 ml Eppendorf tubes kept in dry-ice and pre-filled with two 3mm diameter metal beads. After that Extraction protocol D was performed as described above in all different plant materials simultaneously. For the generation of the pooled extracts, 0.5 ml of the different extracts were pooled to a final volume of 2.5 ml of pooled extract while to standardize the dilution factors, only 2.5 ml of control and treatment extracts were used. After this, the untreated extract, the different treatment (N-starvation, P-starvation, flagellin 22 and chitin) extracts and the pooled extracts which also served as a pooled QC in this study, were dried under a mild stream of nitrogen and stored at -80°C until analysis.

### 2.6 Liquid chromatography mass spectrometry analysis

Three distinct analytical methods were employed to compare the four extraction protocols, analyze specific target metabolites in toxicity assays, and conduct the in-depth metabolic profiling of the extract and comparison of the a/biotic treatments.

#### 2.6.1 Comparison of extraction protocols

Pellets were resuspended in 2 ml of 50% MeOH. After 20-fold dilution samples were filtered with 0.22 μm syringe filters (PVDF) and subjected to analysis. Separation was achieved on a Dionex UltiMate 3000 Quaternary Rapid Separation UHPLC+ focused system (Thermo Fisher Scientific, Germering, Germany) equipped with a ZORBAX Eclipse 1.8 μm XDB-C18 column (100 × 3 mm, 1.8 μm, Agilent). For eluting 0.05% (v/v) formic acid in H_2_O and 0.05% (v/v) formic acid in MeCN were employed as mobile phases A and B, respectively. Gradient conditions were as follows: 0.0−1.0 min 3% B; 1.0−14.0 min 3−20% B; 14.0−20.0 min 20−45% B, 20.0−24.5 min 45−100% B, 24.5−26.5 min 100% B, 26.5−26.55 min 100−3% B, and 26.55−30.0 min 3% B. The flow rate of the mobile phase was 400 μL/min and injection volume were set at 10 μL. The column temperature was maintained at 40°C. UV chromatograms were acquired at 229, 260, 310, and 345 nm. The UHPLC was coupled to a Compact micro-TOF-Q mass spectrometer (Bruker, Bremen, Germany) equipped with an electrospray ion source (ESI) operated in positive or negative ionization mode. The ion spray voltage was maintained at 3750 in positive and -4000 V in negative ionization mode. The dry temperature was set to 275 (positive) and 325°C (negative), and the dry gas flow was set to 8 L/min. Nitrogen was used as the dry gas, nebulizing gas, and collision gas. The nebulizing gas was set to 2.5 bar and collision energy to 15 eV. HRESIMS and MS/MS spectra were acquired in an m/z range from 50 to 1200 amu at a sampling rate of 2 Hz. Sodium formate clusters were used for mass calibration. All files were automatically calibrated by postprocessing. All acquired chromatograms and spectra were analyzed with Compass DataAnalysis v.4.3.0 (Bruker, Bremen, Germany).

#### 2.6.2 Targeted analysis with QQQ for toxicity/permeation assays

Samples were 10-fold diluted with 20% MeOH and subjected to analysis by liquid chromatography coupled to mass spectrometry. Chromatography was performed on an Advance UHPLC system (Bruker, Bremen, Germany). Separation was achieved on a Kinetex 1.7u XB-C18 column (100 x 2.1 mm, 1.7 µm, 100 Å, Phenomenex, Torrance, CA, USA). Formic acid (0.05%) in water and acetonitrile (supplied with 0.05% formic acid) were employed as mobile phases A and B respectively. The elution profile was: 0-0.5 min, 2% B; 0.5-1.2 min, 2-30% B; 1.2-2.0 min 30-100% B, 2.0-2.5 min 100% B, 2.5-2.6 min, 100-2% B and 2.6–4 min 2% B. The mobile phase flow rate was 400 µl min-1. The column temperature was maintained at 40°C. The liquid chromatography was coupled to an EVOQ Elite TripleQuad mass spectrometer (Bruker, Bremen, Germany) equipped with an electrospray ion source (ESI) operated in positive ion mode. The instrument parameters were optimized by infusion experiments with pure standards. The ion spray voltage was maintained at +3500 V. Cone temperature was set to 350°C and cone gas to 20. Heated probe temperature was set to 400°C and probe gas flow to 40 psi. Nebulizing gas was set to 60 psi and collision gas to 1.5 mTorr. Nitrogen was used as probe and nebulizing gas and argon as collision gas. Active exhaust was constantly on. Multiple Reaction Monitoring (MRM) was used to monitor analyte precursor ion → fragment ion transitions. For NMOI3M the detected transitions were 477.3→259.0 and 477.3→97.0 in a retention time of 2.73. Both Q1 and Q3 quadrupoles were maintained at unit resolution. Bruker MS Workstation software (Version 8.2.1, Bruker, Bremen, Germany) was used for data acquisition and processing. Linearity in ionization efficiencies were verified by analyzing dilution series. p-hydroxybenzyl glucosinolate (pOHB) was used as internal standard (Crocoll et al., 2016).

#### 2.6.3 Metabolic profiling of pooled extract and comparison of abiotic and biotic treatments

The metabolite residue was dissolved in 3 ml of 80% MeOH containing 2.5 μM lidocaine, 2.5 μM 10-camphour sulfonic acid, and 10.4 ppm acetic acid. Then, 150 μl of the re-extraction was loaded and eluted with an HLB μElution plate (Waters) that had been conditioned with 200 μl of MeOH and equilibrated with 200 μl of 80% MeOH containing 10.4 ppm acetic acid. The extracts (1 μl) were subsequently analyzed using LC-QTOF-MS (LC, Waters Acquity UPLC system; MS, Waters Xevo G2 Q-Tof). Analytical conditions were as follows LC: column, Acquity bridged ethyl hybrid (BEH) C18 (1.7 μm, 2.1 mm * 100 mm, Waters); solvent system, solvent A (water including 0.1% [v/v] formic acid) and solvent B (acetonitrile including 0.1% [v/v] formic acid); gradient program, 99.5%A/0.5%B at 0 min, 99.5%A/0.5%B at 0.1 min, 20%A/80%B at 10 min, 0.5%A/99.5%B at 10.1 min, 0.5%A/99.5%B at 12.0 min, 99.5%A/0.5%B at 12.1 min and 99.5%A/0.5%B at 15.0 min; flow rate, 0.3 ml/min at 0 min, 0.3 ml/min at 10 min, 0.4 ml/min at 10.1 min, 0.4 ml/min at 14.4 min and 0.3 ml/min at 14.5 min; column temperature, 40°C; MS detection: polarity, positive/negative; capillary voltage, +3.00 kV (positive)/-2.75 kV (negative); cone voltage, 25.0 V; source temperature, 120°C; desolvation temperature, 450°C; cone gas flow, 50 l/h; desolvation gas flow, 800 l/h; collision energy, 6 V; mass range, m/z 50–1500; scan duration, 0.1 sec; interscan delay, 0.014 sec; data acquisition, centroid mode; Lockspray (Leucine enkephalin); scan duration, 1.0 sec; interscan delay, 0.1 sec. MS/MS data was acquired in the ramp mode as the following analytical conditions: (1) MS: polarity, positive/negative; mass range, m/z 50–1500; scan duration, 0.1 sec; inter-scan delay, 0.014 sec; data acquisition, centroid mode and (2) MS/MS: polarity, positive/negative; mass range, m/z 50–1500; scan duration, 0.02 or 0.1 sec; inter-scan delay, 0.014 sec; data acquisition, centroid mode; collision energy, ramped from 10 to 50 V. In this mode, MS/MS spectra of the top 10 ions (> 1000 counts) in an MS scan were automatically obtained. If the ion intensity was less than 1000, MS/MS data acquisition was not performed and moved to of next top 10 ions.

### 2.7 Pre-processing with MSDIAL

After LC-MS analysis the acquired data were introduced to MSDIAL 4.0 (Tsugawa et al., 2020) for pre-processing which included the following steps: peak picking, deconvolution, peak alignment and peak identification. The datafiles acquired on the Bruker Compact microToF were initially transformed into.mzMl files with an in-house script and the precursor m/z values were subsequently corrected with a published script (Breaud et al., 2022) to address a well-known issue occurring during Data-Depended Analysis in some Bruker instruments. The datafiles generated from the Waters Xevo Q-ToF were introduced in MSDIAL directly for analysis. After pre-processing all datasets were filtered within the MSDIAL environment with the use of MS-CleanR (Fraisier-Vannier et al., 2020) to remove non-informative metabolic features based on the following criteria: Minimum blank ratio: 0.8, removal of ghost peaks, maximum Relative Standard Deviation (RSD) 30%. The comparison of the tested extraction protocols was performed by aligning each extraction protocol with their respective blank and exporting them as individual datasets for further analysis. The impact of the different artificially induced treatments was assessed by aligning all different treatments in a common dataset, which was exported for statistical analysis.

### 2.8 Statistical analysis with graphPad prism, metaboAnalyst and R

#### 2.8.1 Comparison of different extraction protocols

After export from MSDIAL the four different extraction protocols were introduced to GraphPad Prism for analysis (**Supplementary File 1**). For the intraday precision, the Relative Standard Deviation (RSD) of all metabolic features per protocol after filtration was initially calculated among different technical replicates (n=5) followed by the determination of the median RSD per protocol. For the determination of the interday precision, we calculated the Pooled Relative Standard Deviation (PRSD) among three different days (day 1, day 20 and day 35) of measurements for all individual metabolic features (n=5/day of measurement) as well as the median PRSD per extraction protocol based on the following formula:


(1) $$\text{PRSD}=\sqrt{\frac{({\text{n}}_{1\;}-1){\text{RSD}}_{1}^{\;2}+\;({\text{n}}_{2\;}-1){\text{RSD}}_{2}^{\;2}+\ldots +({\text{n}}_{\text{k }}-1){\text{RSD}}_{\text{k}}^{\;2}}{{\text{n}}_{1\;}+{\text{n}}_{2}+\ldots +{\text{n}}_{\text{k }}-\text{k}}}$$

, where n represents the number of measurements in each individual day and RSD represents the Relative Standard Deviation of the metabolic feature within each day of measurements (Doppler et al., 2016). The same technical replicates were measured throughout the different days of measurement after storage at -80°C (n-total = 15). A one-way ANOVA was performed on the median RSD, followed by a Tukey post-hoc test to determine any significant differences among the median RSD and median PRSD, respectively.

#### 2.8.2 Comparison of artificially induced treatments and distinguishment of treatment-specific metabolites

For the investigation of the different treatments, we used a combination of MetaboAnalyst (v.6.0) and the relevant R package MetaboAnalystR for statistical analysis. After export the data (one dataset per ionization mode) were filtered and all metabolic features presenting RSD ≥ 30% in the pooled QC samples were removed (**Supplementary Table 2**, **Supplementary Table 3**). For multivariate analysis, the data were normalized against the internal standard, log2 transformed and Pareto scaled to achieve a closer to normal distribution of the values. After that, an unsupervised Principal Component Analysis (PCA) was performed on the transformed data followed by hierarchical cluster and heatmap analysis on the identified metabolites to identify treatment-specific metabolic patterns. The Hierarchical Cluster Analysis (HCA) was calculated using Euclidean distance and ward-linkage.

The comparison of the individual treatments and the pooled extract against the control (untreated) extract was based on multiple t-testing after normalization of the data against the internal standard. The minimum criteria for considering a metabolic feature as significantly different among the tested group and the control was a significant threshold of p ≤ o.o5 and a mean-fold change of 2 between the respective groups. The p values were corrected based on the False Discovery Rate for multiple testing to reduce the number of false positives (**Supplementary File 2**).

### 2.9 Metabolite identification and spectral similarity networking

Metabolite identification was conducted as described previously (Tsugawa et al., 2019) without merging of positive and negative ionization modes. For level 1 identities, the m/z and retention time of metabolic features were matched against pure metabolite standards included in PlaSMA database. Level 2 identifications were achieved by matching experimental MS_2_ spectra against reference MS_2_ spectra in various MS/MS spectral databases (MetaboBASE, GNPS, MassBank, Metlin, ReSpect, Nist14 etc.). If no structure was found, the metabolic features were introduced to MS-FINDER and their chemical formulas were calculated and ranked. After ranking another database search based on the calculated formulas was conducted against multiple databases (KNApSAck, UNPD, PlantCyc, FoodDB, NANPDB, STOFF-IDENT, LipidMAPS, HMDB, PubChem, SMPDB, YMDB, ECMDB, BMDB, TSDB, Drugbank) and structure candidates were retrieved (Level 3.1). If no candidate structures were found, Fragment Set Enrichment Analysis (FSEA) was conducted with MS-FINDER to obtain metabolite class (ontology) information (Level 3.2), (**Supplementary Method 1**). The table including information from all identification levels was used to generate the molecular networks based on MS/MS similarity. Edges were generated when two features presented a minimum peak match of 6; match threshold of 0.8; and a max number of edges per node of 10 was allowed.

## 3 Results

### 3.1 Design of extraction protocols

**Figure 1:**
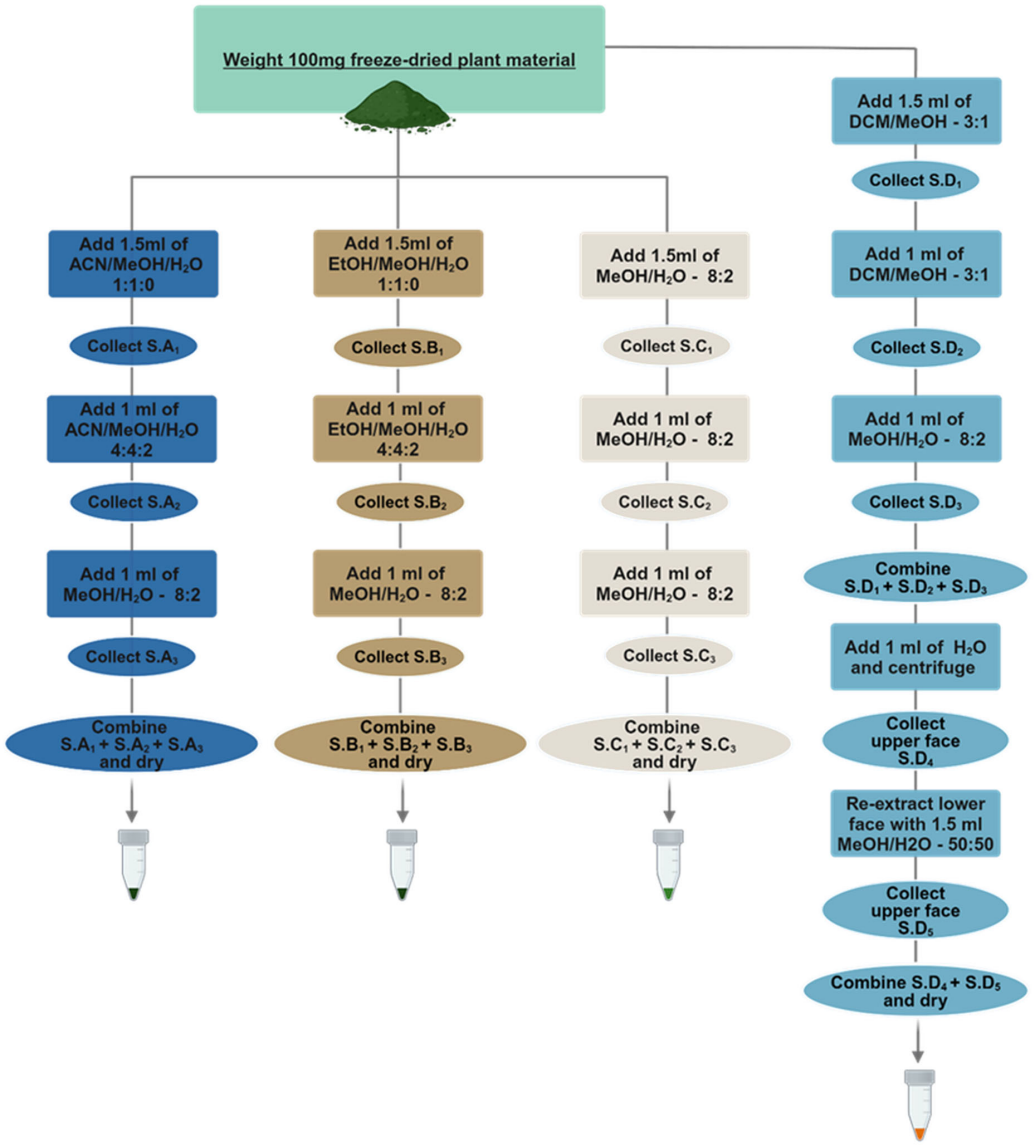
Schematic representation of the four different extraction protocols. Protocol A-Acetonitrile/Methanol/Water (ACN/MeOH/H_2_O), B-Ethanol/Methanol/Water (EtOH/MeOH/H_2_O) are one-phase protocols and make use of different extraction solvent mixtures in the different extraction steps starting from the more non-polar solvents towards the more polar mixtures. Protocol C-Methanol/Water (MeOH/H_2_O) makes use of the same one phase extraction solvent throughout the whole process. Protocol D-Dichloromethane/Methanol/Water (DCM/MeOH/H_2_O) is a two-phase Liquid-Liquid Extraction protocol where only the upper phase is being used downstream for analysis while the lower phase is discarded. Abbreviation S. corresponds to supernatant.

To create a comprehensive extraction protocol with maximized coverage and compatible with transport assays in Xenopus oocytes, we investigated four extraction protocols (A, B, C and D) composed of different extraction solvent mixtures commonly used in metabolomics studies in varying ratios (**Figure 1**).

The conceptualization of the first two extraction protocols A (Acetonitrile/Methanol/Water) and B (Ethanol/Methanol/Water) was based on the idea that alternating the polarity of each extraction step, starting from lipophilic towards more hydrophilic solvents, would expand the metabolic space captured. The order of the solvents was adopted from sequential and accelerated solvent extraction methodologies (Chamali et al., 2023; Gil-Martín et al., 2022; Hajji Nabih et al., 2023; Palaiogiannis et al., 2023) and aimed at defatting the plant material in the first extraction step to enhance the efficiency of subsequent extractions, by increasing the diffusivity of the solvent to the plant material and allow the solvent to release matrix-bound metabolites. Extraction protocol C was adapted from (De Vos et al., 2007; Crocoll et al., 2016) since it has been commonly used for metabolomic studies in Arabidopsis, while the liquid-liquid extraction protocol D was developed as an alternative to the Matyash (Matyash et al., 2008) and Bligh-Dyer (Bligh and Dyer, 1959) methods, since it allowed the simultaneous extraction of metabolites and removal of pigments, lipids and interfering compounds. In all four protocols, all extraction steps of the plant material maintained a high amount of organic solvent (80% and above) to avoid enzymatic degradation of metabolites and included sonication steps to enhance the extraction process. (t’Kindt et al., 2009; Masson et al., 2010; Tulipani et al., 2013; Bijttebier et al., 2016; Salem et al., 2016; Sostare et al., 2018; Schippers et al., 2023; Zenati et al., 2023).

### 3.2 Comparison of extraction protocols metabolic content

**Figure 2:**
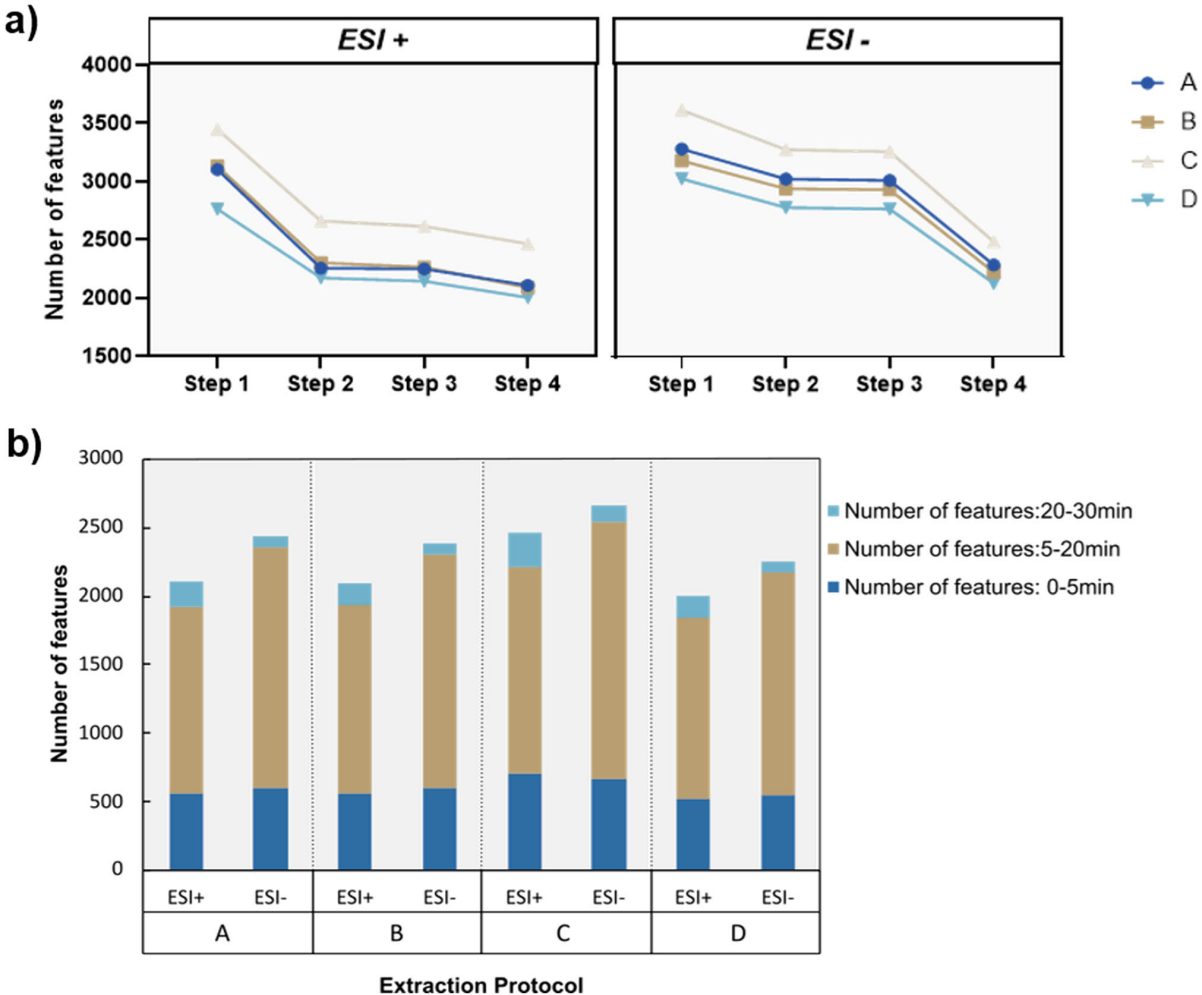
Evaluation of metabolic features captured by each protocol. **(a)** Number of metabolic features after each consecutive filtering step. Step 1: Features after alignment and gap filling. Step 2: Features after removal of peaks with a height ratio between samples and blanks ≤ 0.8. Step 3: Features after removal of peaks with significant retention time drift. Step 4: Features after removal of peaks with a Relative Standard Deviation ≥ 30%. **(b)** Number of metabolic features for each extraction protocol in the different ionization modes. Stack bars highlight the number of features binned in the retention time dimension which is an estimate for the proportion of polar (0-5 minutes), semi-polar (5-20 minutes) and lipophilic (20-30 minutes) molecules in each extraction protocol. (n = 5 technical replicates/protocol).

For the selection of extracts as substrate for oocyte-based transport assays, we used different criteria to evaluate which extraction protocol is more appropriate for this experimental set-up. As a first step, we evaluated the number of metabolites captured in each extraction protocol by comparing the amount and quality of metabolic features detected among five technical replicates. To reduce the number of background metabolic features, we applied extensive feature filtering with MsCleanR (Fraisier-Vannier et al., 2020) and removed features present in the extraction blanks arising from the different solvent mixtures, as well as features with high background ion drift in samples where there was a significant shift in the average retention time of the features. As a final-filtration step, we used the Relative Standard Deviation (RSD) of each metabolic feature detected among replicate measurements as an estimate of the reproducibility of detection of the individual features. Based on this criterion only features with a RSD ≤ 30% were considered for downstream analysis (**Figure 2a**).

For both ionization modes (positive and negative Electrospray ionization (ESI)) a similar trend was observed, where the initial filtration step against blanks had the highest impact on the number of metabolic features filtered out of the dataset. The first filtering step against the blanks had a more profound effect on extraction protocols A and B that make use of the more lipophilic solvents acetonitrile and ethanol. This was observed specifically during ESI+ where 26.4% (protocol A) and 26.4% (protocol B) of total detected features were removed. We suspect that this tendency is due to the heavy load of strongly retained lipophilic compounds, contaminants such as plasticizers and other commonly found impurities and artifacts, arising during the extraction process that will be partitioned heavily in more non-polar systems due to their octanol/water partition co-efficient (logD) values.

Regarding the sheer number of metabolic features (**Figure 2b**), protocol C outperforms the other three protocols since it contains the highest metabolic load in both ionization modes with 2466 (ESI+) and 2676 (ESI-) metabolic features, while protocol D presents the lowest number of features with 2005 (ESI+) and 2251 (ESI-) metabolic features detected after filtration. This was an expected outcome considering that in protocol D, a substantial proportion of metabolites with increased lipophilicity including pigments and plant-related lipids have mostly partitioned to the Dichloromethane lower phase and therefore excluded from the analysis. Next, we evaluated the distribution of the different metabolic features detected across the retention time. Since we use a Reversed Phase C-18 column, we expect that an extraction protocol with more features detected during the late elution phase (20–30 minutes) will be enriched with more non-polar compounds. In the context of oocyte-based transporter assays, pigments, lipids and lipid-like molecules are considered interfering compounds since they may have the ability to permeate through the oocyte membrane and compromise the health of the oocytes. In addition, permeating compounds may produce false positives and be recognized as substrates for specific transporter proteins, while their overaccumulation inside the heterologous expression system is the outcome of passive diffusion. Therefore, we consider an increased content of lipophilic metabolites as a negative attribute of the extraction protocols. In that regard, protocols A and C perform worse than the other two since they present the highest number of metabolic features between 20–30 minutes, while protocols B and D present the lowest proportion of late eluting features.

### 3.3 Evaluation of natural RSD distribution, intra- and interday precision

**Figure 3:**
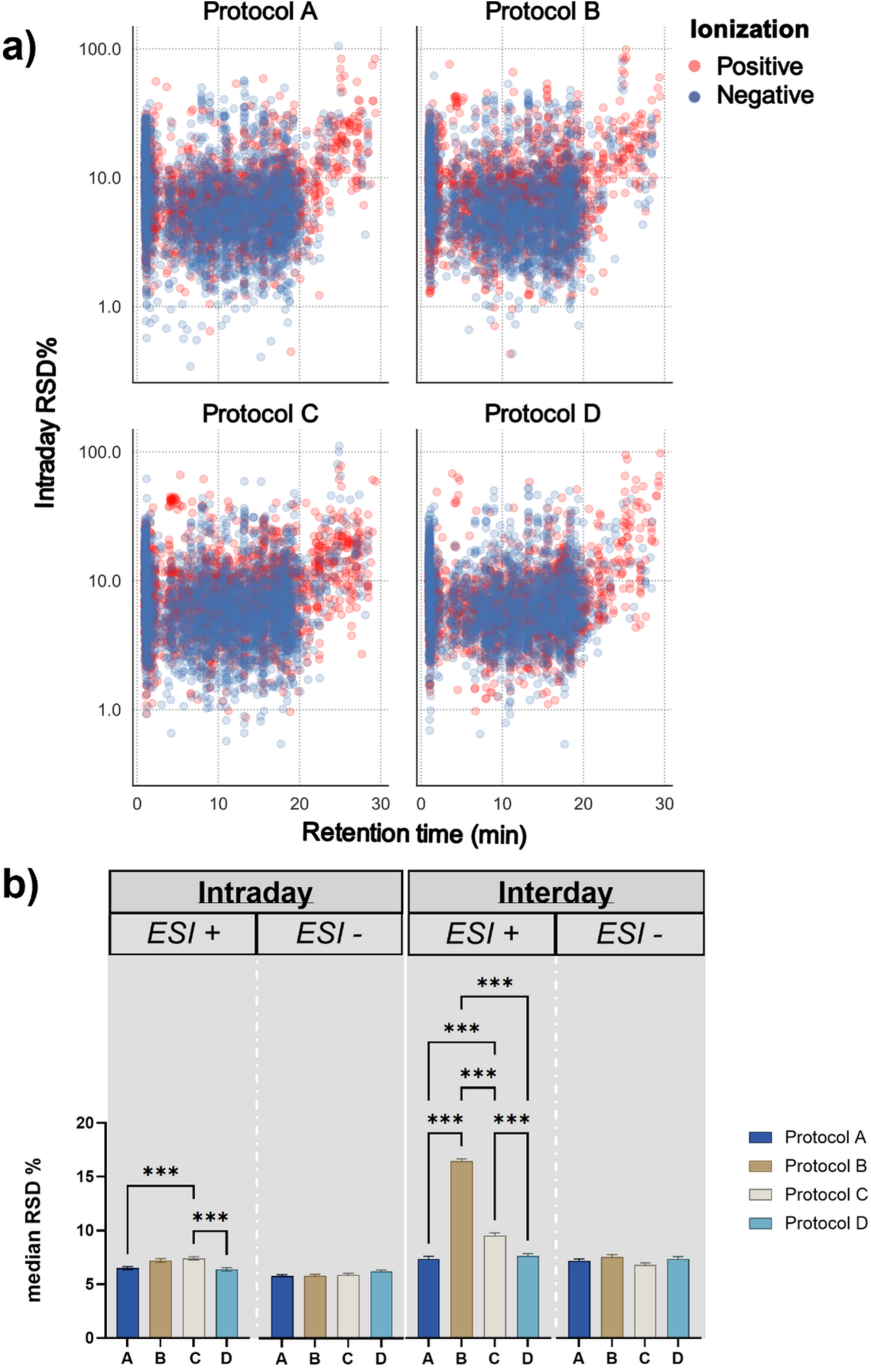
Comparison of analytical performance of the different extraction protocols. **(a)** Distribution of the individual metabolic feature Relative Standard Deviations among their respective retention time for the four different extraction protocols in both ionization modes. ESI+: positive Electron Spray Ionization. ESI-: negative Electron Spray Ionization **(b)** One-way ANOVA with Tukey´s post-hoc test for the comparison of the intraday (n = 5) and interday precision (n = 15) among the four different extraction protocols. For significantly different median RSDs *** represents p ≤ 0.001 for post-hoc Tukey analysis.

As a next step, the four different extraction protocols were evaluated with respect to the distribution of the RSD of the individual detected features and their intra- and interday precision (**Equation 1**), to get an estimate of how similarly the extracts behave. To estimate how consistently metabolic features were detected in each extraction protocol, and how similarly individual features behave throughout the same day of measurements, we plotted the Relative Standard Deviation of the individual metabolic features detected in each protocol prior to any filtering with MsCleanR. This enabled us to investigate the natural variation of the four different protocols and identify specific patterns that could assist us on the selection of the best performing extraction. Irrespectively of the extraction protocol assessed, the metabolic features detected in positive ionization present slightly elevated RSDs in comparison to the features detected in negative ionization (**Figure 3a**). Additionally, we observed increased RSDs of the metabolic features in the beginning of the gradient where most of the non-retained metabolites elute, as well as an increasing tendency in the RSDs of metabolic features detected after minute 20 along the gradient. This demonstrates that regardless of the extraction protocol used, the metabolic features detected in the beginning and by the end of the method, exhibit reduced reproducibility among replicate measurements. Noticeably, RSDs of features detected in protocol D, present a distinctively lower variation and a more uniform distribution of their RSD values the majority of which ranges between 1 and 30%.

For the estimation of the intraday precision, we used the median Relative Standard Deviation of metabolic features detected among replicates in each protocol (**Equation 1**) after the filtering with MsCleanR (**Figure 3b**). Since the metabolic features with RSD ≥ 30% have been removed, the median RSD of all different extraction protocols was lower than 10%. Interestingly, in *ESI+* protocol C presented a significantly lower intraday precision compared to protocols A and D that exhibited the best performance with a median RSD of 6.48 and 6.37% respectively, while no significant differences were observed among the different protocols for features detected in ESI-. The interday precision, expressed as median pooled RSD, was calculated based on measurements of the exact same samples in three randomly selected days (day 1, day 20 and day 35) and was used as a metric to understand the extent of day-to-day variability under the same experimental conditions. Among the four different protocols, protocol B presented a significantly higher day-to-day variation in ESI+ when compared to the other three protocols with a median pooled RSD of 16.4%, followed by protocol C which also presented elevated interday pooled RSD of 9.5%. Similarly to intraday results, protocols A and D presented the lowest variation with 7.34 and 7.6%, respectively. No significant differences were observed for the interday precision among the four extraction protocols in the negative ionization mode. Based on these results we concluded that, regarding both intra- and interday precision, extraction protocols A and D outperform the other two protocols and give slightly more reproducible results, particularly for metabolic features detected in positive ionization mode. In addition, the distribution of the Relative Standard Deviation of the individual features prior to any dataset clean-up, reveals a smaller variation on the RSD values of the features included in protocol D besides the considerably more complex experimental set-up.

### 3.4 Evaluation of toxicity towards Xenopus oocytes based on permeation assays

We evaluated the membrane permeating capabilities of the two most prominent extraction extracts isolated using protocols C and D. Protocol C was selected as it results in the most metabolic features after filtration despite presenting a lower precision compared to protocol A. Protocol D was selected due to the high intra- and interday precision and its capability to remove lipophilic compounds and pigments that are likely to compromise the health of the *Xenopus* oocytes (Maldifassi et al., 2016). For the toxicity assay, the extract pellets were resuspended and diluted with pH=5 Kulori buffer to ensure similar pH along the assay and prevent osmotic eruption of the oocytes. To uniformly resuspend metabolites from the extract pellets, we complemented the buffer with 5% ethanol, aiming to bring the polarity of the resuspension solvent closer to the polarity of the extraction solvents used (Lindahl et al., 2017). For the assay, the oocytes were incubated at room temperature for one hour in different extract dilutions, washed multiple times to remove remnants of the surrounding extract, and subsequently homogenized for analysis in a final concentration of 20% methanol solution.

To evaluate permeation of the plasma membrane of oocytes, we tested increasing dilutions of resuspended pellets of lyophilized extracts generated from both extraction protocols and measured the presence of selected non-permeating metabolites within the oocyte. As an example, we used the sulphated specialized metabolite 1-methoxy-3-indolylmethyl glucosinolate (NMOI3M), which is one of the most abundant glucosinolates found in young *Arabidopsis* seedlings (Crocoll et al., 2016). In our experimental set-up, absence of NMOI3M was indicative of preserved membrane integrity, while detection of NMOI3M signified plasma membrane permeation.

**Figure 4:**
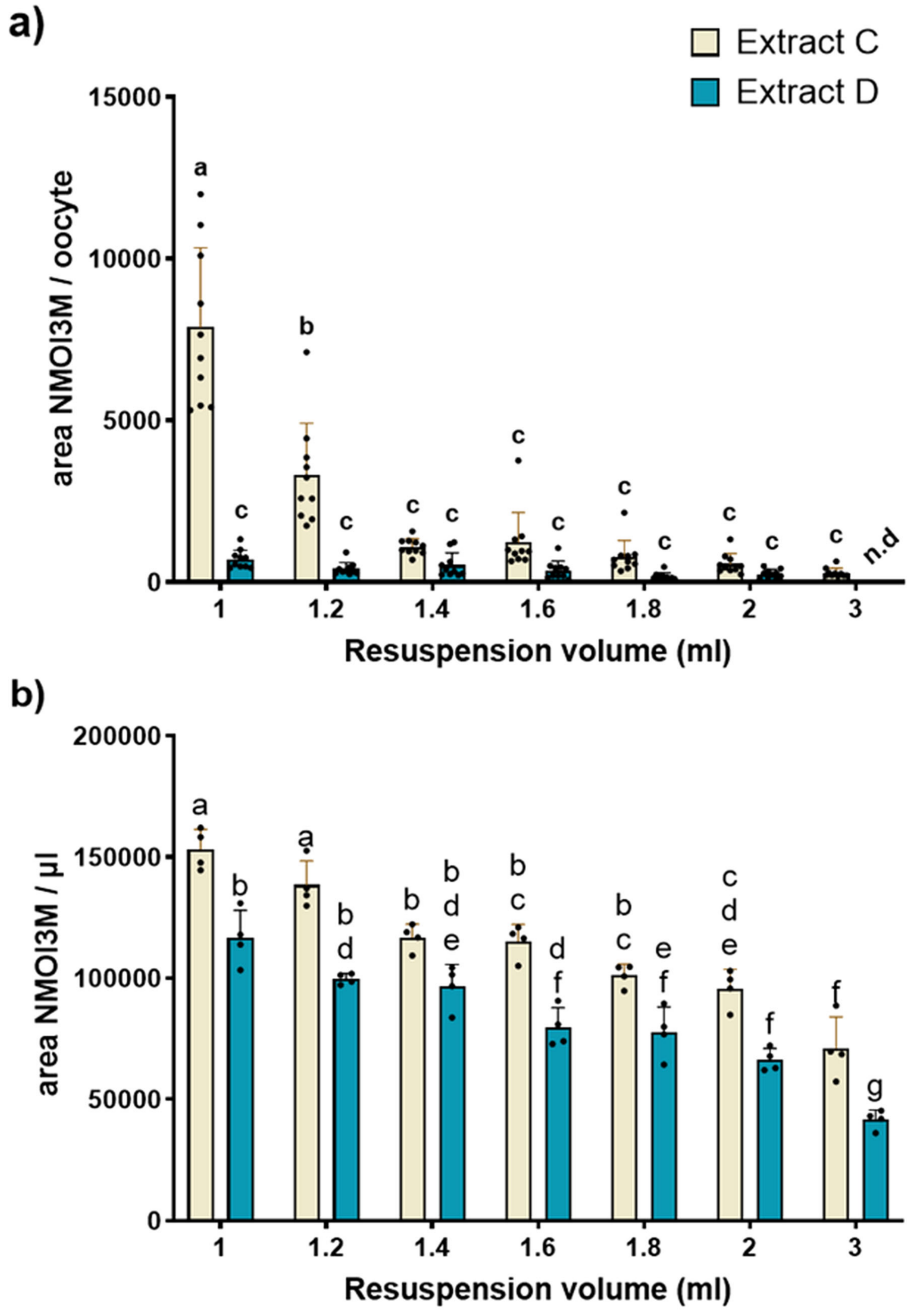
Permeation assay results for the two most prominent extraction protocols. **(a)** Comparison of the concentration of NMOI3M between extraction protocol C and D inside the oocytes for the different dilution levels. Individual oocytes were assayed with different media formulations of C and D and analysed for Neoglucobrassicin (NMOI3M) presence (n=10). Two-way ANOVA with Tukey’s multiple comparison correction. Different letters represent statistical differences. Detection of the non-permeating NMOI3M indicates permeation on the tested concentration. **(b)** Relative levels of NMOI3M of the two tested extracts in the tested dilutions (n = 4). Abbreviation n.d corresponds to values below method’s LoD.

For the selection of NMOI3M as a permeation marker, we modelled the different charged states (anion vs. protonated/uncharged) of NMOI3M and calculated its logD value in pH=5 where the permeation assays take place (**Supplementary Figure 2**). As expected, NMOI3M remains completely in the anion state and presents a negative logD value of -3.10 indicating high hydrophilic properties. We concluded that NMOI3M is an excellent compound for permeation assays and that detection of the compound within the oocyte homogenate would signify membrane disruption. We observe that, oocytes exposed to the highest concentrations of extracts formulated with protocol C display a higher amount of permeation than oocytes exposed to their protocol D counterparts (**Figure 4a**). Specifically, for smaller dilutions (1 and 1.2 ml), the concentration of NMOI3M increased significantly in oocytes assayed with protocol C in comparison to protocol D. For resuspension volumes of 1.4 ml and above, oocytes assayed with protocol C exhibited higher levels of NMOI3M compared to oocytes assayed with protocol D although differences were not statistically significant. For the highest dilution of 3 ml, no NMOI3M signal was detected for protocol D, while some NMOI3M was still detected within the oocytes exposed to protocol C. These results clearly demonstrate that extraction protocol D presents reduced permeation towards the oocyte compared to protocol C, where a higher degree of dilution was required to mediate the observed permeation. As an alternative, we tested Kulori buffer supplemented with 5% DMSO to assess the effect of different organic solvents to oocyte membrane integrity which we rejected due to increased toxicity (**Supplementary Figure 3**).

To confirm the detectability of NMOI3M across all tested dilutions and to determine whether the observed differences between the two protocols resulted from enhanced permeation, we analyzed the varying levels of NMOI3M captured by each protocol. (**Figure 4b**). Indeed, NMOI3M was detected even in the highest tested dilutions (3 ml), indicating that absence from the oocytes assayed with extract D was due to their intact plasma membrane. Regarding the relative levels of NMOI3M between the two extraction protocols, C presented an increased amount of NMOI3M compared to D in all tested dilutions. Although these differences were significant and may partly explain the variations in permeation assay results observed in oocytes tested with different protocols, they do not account for the drastic reduction of the NMOI3M signal in oocytes exposed to the smallest dilutions (1 and 1.2 ml) of extract D. This clearly shows that the differences in NMOI3M levels observed in oocytes tested with different protocols were not due to variations in NMOI3M levels between extracts C and D but rather to differences in the extracts’ ability to permeate the oocyte membranes.

**Figure 5:**
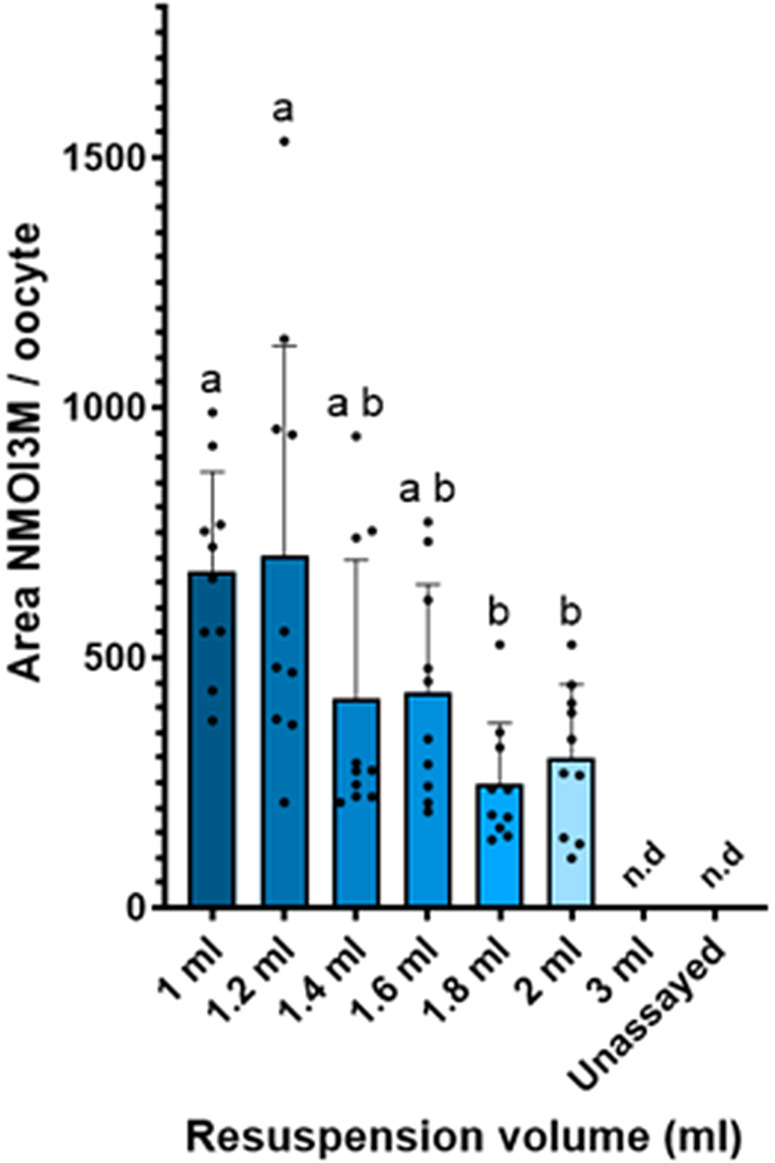
Relative levels of NMOI3M after assaying oocytes with different dilution degrees of extract D. Individual oocytes were assayed with different media formulations of extracts from protocol D and analyzed for NMOI3M presence. n=10. One-way ANOVA with Tukey’s multiple comparison correction. Different letters represent statistical differences. Un-assayed oocytes not exposed to plant extracts were used as a background reference. Abbreviation n.d corresponds to values below LoD.

Next, we looked at the concentrations of NMOI3M between oocytes assayed with extract from protocol D in different dilutions to determine if any significant differences are observed and to find the appropriate dilution for oocyte-based screening assays. With increasing dilution, the concentrations of NMOI3M were reduced and significantly differed among various levels (**Figure 5**). The NMOI3M levels of oocytes assayed with the highest concentrations of protocol D-derived extracts displayed a high variability. The increased RSD among replicates might be due to the closeness of the measurements to the method’s Limit of Detection.

### 3.5 Evaluation of the impact of treatments on the metabolic diversity

**Figure 6:**
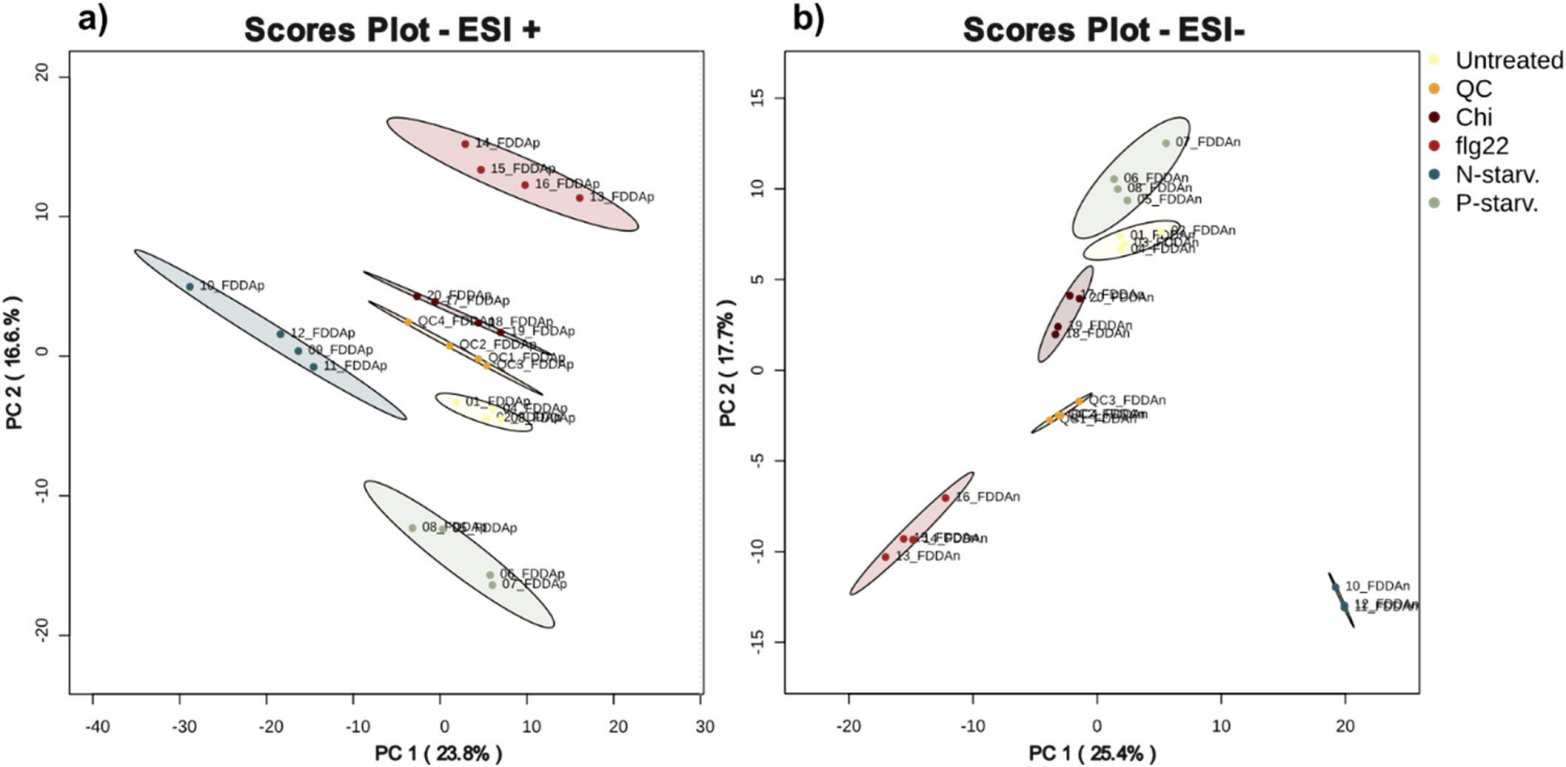
Principal Component Analysis of the different treatments when analysed in **(a)** positive and **(b)** negative ionization mode. For both modes the pooled extracts (QC samples) cluster in the middle of the different groups.

After selecting the appropriate extraction protocol (protocol D) we investigated the impact of the different biotic and abiotic treatments on the metabolic diversity captured on the final extract. As a first step, we performed an unsupervised Principal Component Analysis to look if the different treatments could be separated from one another. In this study, the pooled extracts generated by combining extracts from treated and untreated plants in equal amounts also served the purpose of pooled Quality Control (QC) samples. The scores plots (**Figure 6**) of the first two Principal Components were able to explain 40.4% and 43.1% (sum of PC1 and PC2) of the total observed variance for ESI+ and ESI- respectively, indicating distinct separation of the different treatments and an overall altered metabolic diversity among them (**Figure 6**).

**Figure 7:**
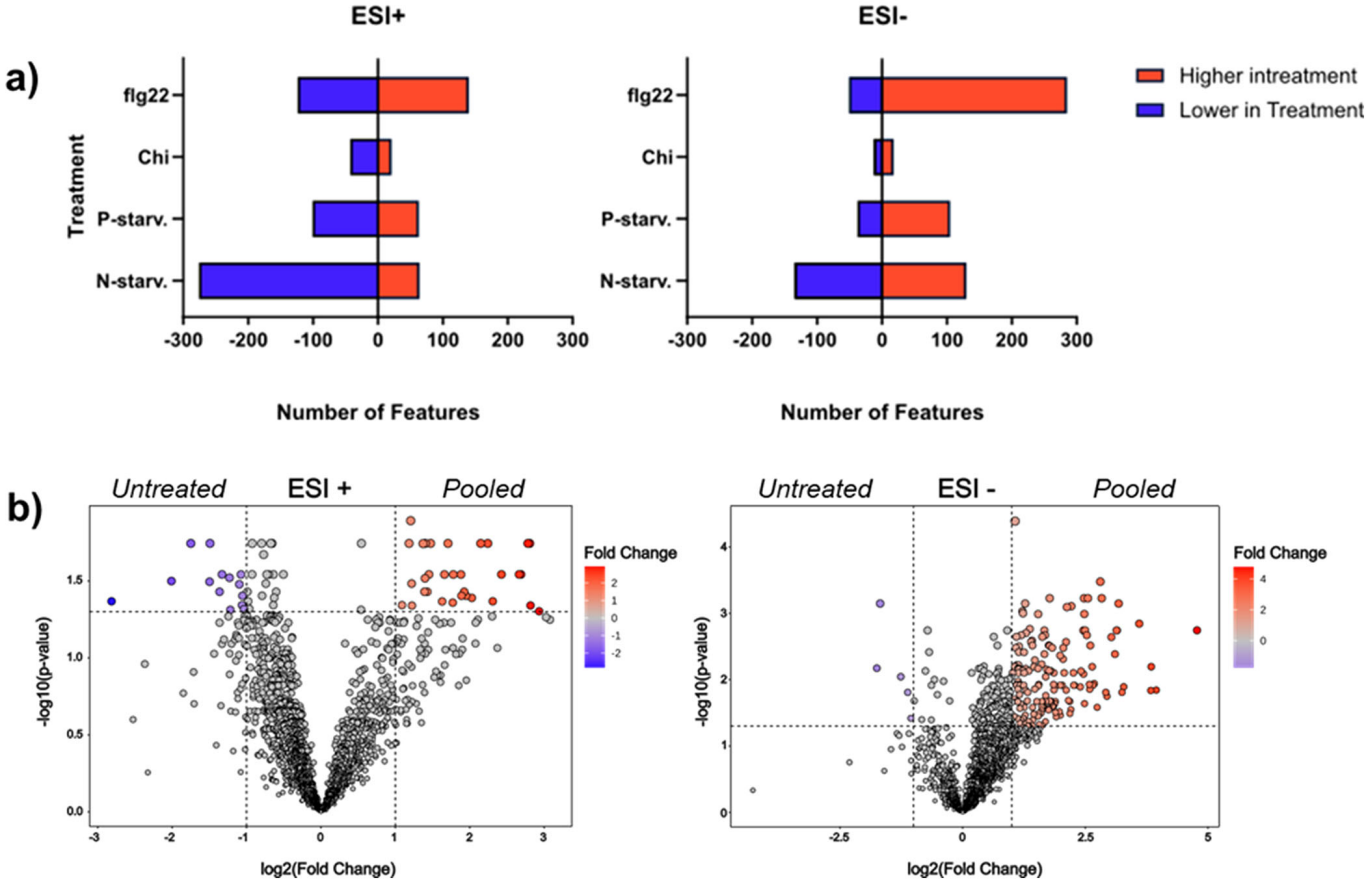
Evaluation of the impact of different treatments om the metabolome. **(a)** Number of features significantly altered between individual treatments and the control untreated plants in both ionization modes. **(b)** Volcano plots representing the multiple t-test comparisons between the final pooled and the untreated extracts for both ionization modes. In both modes the pooled extracts representing an overview of all treatments, present a higher number of features significantly increased and only a small fraction of metabolic features significantly reduced when compared to untreated plant extracts.

To get an overview of the impact of each treatment on the complexity of the plant metabolome compared to the untreated plants, we conducted pairwise comparisons (treatment x vs untreated) with multiple t-testing for each metabolic feature after correcting their p values with the False Discovery Rate method to minimize the number of false positives. The volcano plots representing cumulative results of all comparisons for both ionization modes can be found in **Supplementary Figure 4**, while the final count of significantly different metabolic features after each comparison can be seen in **Figure 7a**. We observed that in ESI+, for all different treatments except flagellin 22, less features were detected in the treated extracts in comparison with the untreated extract. Noticeably, nitrogen starvation exhibited the most extreme case possibly due to the reduced levels of amino acids. In ESI- most of the treatments except nitrogen starvation showed a higher number of features in comparison to the untreated plants, while in both ionization modes chitin seems to be the treatment that has the least impact on the metabolome with a very small number of metabolic features differentiating between the treatment and the controlled plants. Next, we performed a direct comparison of the pooled extracts versus the untreated plant extract to determine if the pooled extract includes significantly increased metabolites, or the majority of the metabolic features present lower intensities due to the dilution effect of mixing the different extracts. The results show that in both ionization modes more features were significantly increased in the pooled extract than the untreated plant extract (**Figure 7b**). These data verify our assumption that besides the cumulative dilution due to pooling of the different treatments, the final extract is enriched with metabolic features compared to the untreated plant extract.

**Figure 8:**
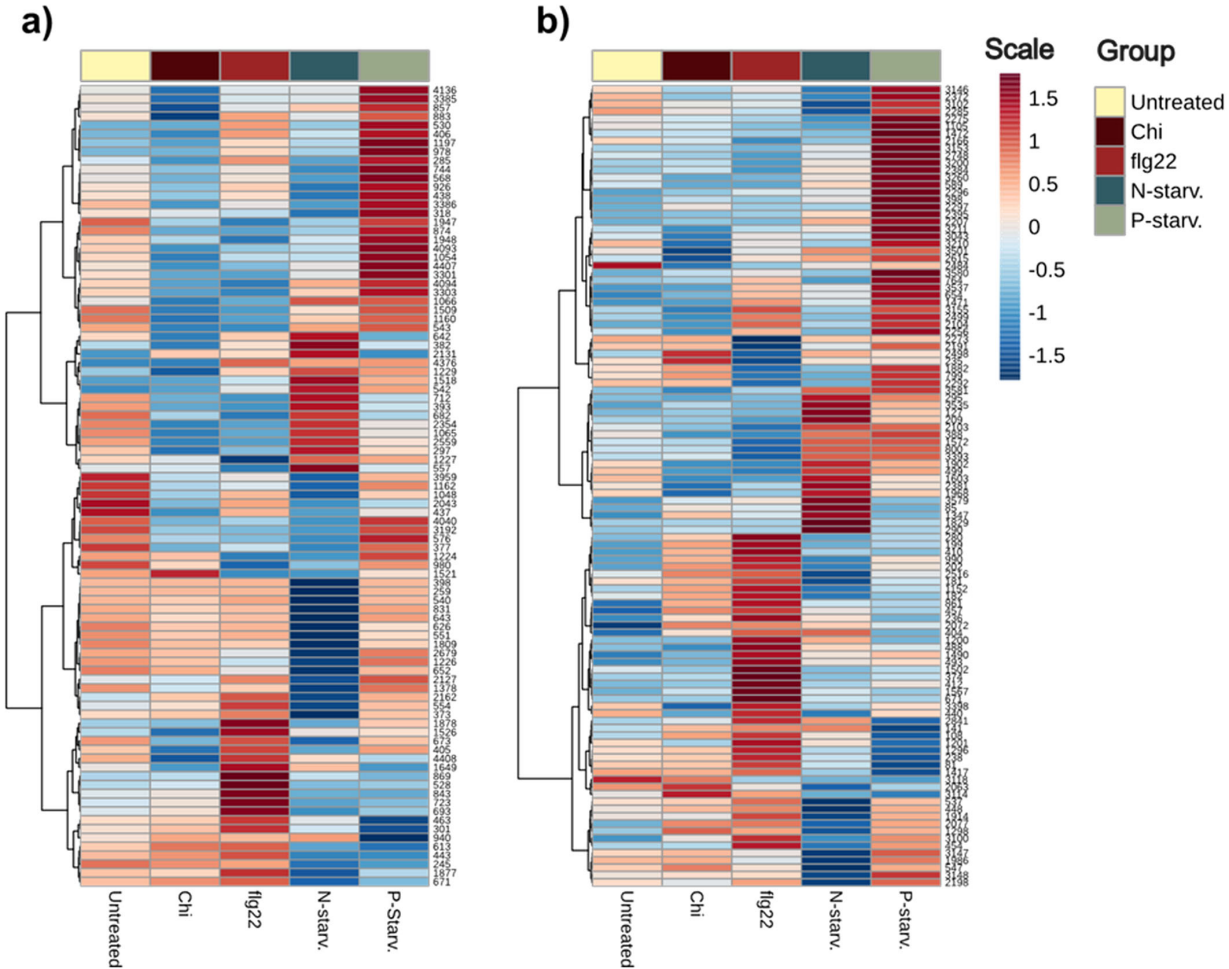
Heatmap of level 1 and 2 identified metabolites from both ionization modes. Hierarchical Clustering has been performed to metabolites to verify the presence of treatment specific metabolites. **(a)** Metabolites identified in Positive ionization (ESI+). **(b)** Metabolites identified in Negative ionization (ESI-).

Next, we investigated specific metabolic patterns induced under the different (a)biotic treatments on the metabolic diversity to establish biomarker metabolites specific to individual treatments. Only metabolites identified with high identification confidence (*levels 1:* Identified against standards and *2:* tentatively annotated against databases based on MS_2_ spectra) were considered (**Supplementary Table 1**). The results presented as heatmaps illustrate that after Hierarchical Cluster Analysis on the identified portion of metabolites, distinct metabolic clusters can be seen for the different treatments. In agreement with our previous results, where all the metabolic features were considered for univariate analysis, flagellin, nitrogen and phosphorus starvation, present the distinct metabolic patterns, where increase or reduction of individual metabolites can be correlated with the different treatments. From all applied treatments, chitin is the one giving the most subtle differences with only a few metabolites correlating with the treatment (**Figure 8**).

Flagellin induced a strong response in the plant metabolome affecting the levels of many metabolites across different metabolic pathways, such as both identified isomers of Coumaroyl agmatine from the Hydroxycinnamic acid amides pathway, 2-amino-9-methanesulfinylnonanoic acid and 5-methanesulfinylpentanenitrile from glucosinolate breakdown metabolites, Indole acetaldehyde, Indole-5-carboxylic acid, 1-methoxyindole-3-carbaldehyde and Indole + 1O, 1carboxy, O-Hex connected to auxin IAA and tryptophan pathways, the amino acids isoleucine and N-Acetyl-D-phenylalanine as well as coniferyl aldehyde from lignin biosynthesis (Bednarek et al., 2005; Lim et al., 2005; Muroi et al., 2009; Sairanen et al., 2012; Tsugawa et al., 2019).

Extracts from nitrogen-starved plants, present one distinct cluster of up-concentrated metabolites, and another indicating reduction of metabolites in comparison to both the other treatments and the untreated plants. Regarding the first cluster, compounds such as the modified and normal amino acids N-Acetyl-L-proline, L-Serine and N-Methyl-D-aspartic acid, reduced and oxidized glutathione, small TCA derived organic acids such as malic and maleic, lysine catabolite pipecolic acid (Wang et al., 2021) and coniferyl alcohol presented an increased concentration in the nitrogen starved plant extracts. On the other hand, the cluster with significantly lower metabolites included nitrogen-containing compounds such as 4-aminobutyl guanidine and spermidine, major amino acids and derivatives including arginine, histidine, pyroglutamic acid, lysine, citrulline and N-Acetyl-glutamine as well as other metabolites like stilbene glycoside trans-piceid, 4-methylthiobutyl and 5-methylthio-n-pentyl glucosinolates and the glycosylated sinapic acid derivative, disinapoyl hexoside.

Extracts from phosphorus-starved plants presented a similar behavior as nitrogen starvation, with both increased and decreased metabolites signifying the success of the treatment. Various classes of metabolites such as glucosinolates (e.g 5-methylsulfinylpentyl, methylsulfinylhexyl, methylsulfanyl heptyl), the majority of different types of flavonoids (e.g Cyanidin 3-2G-glucosylrutinoside, Flavonol base + 3O, 1MeO, O-dHex-dHex, Quercetin-3-O-rhamnoside, Kaempferol-3-O-rhamnoside), as well as both coumaroyl hexoside isomers, were found in the cluster with increased intensity. In contrast, a strong decrease in major phenolic glucosides (Benzoic acid + 1O, O-Hex, Benzoic acid + 2O, O-Pen, Benzoic acid + 2O, O-Hex), the related hormone salicylic acid and phosphorus containing o-phosphocholine was observed. Based on these differences, we can conclude that enriching the final pooled extract with phosphorus starved plants leads to the increase of most identified flavonoids in the extract.

**Figure 9:**
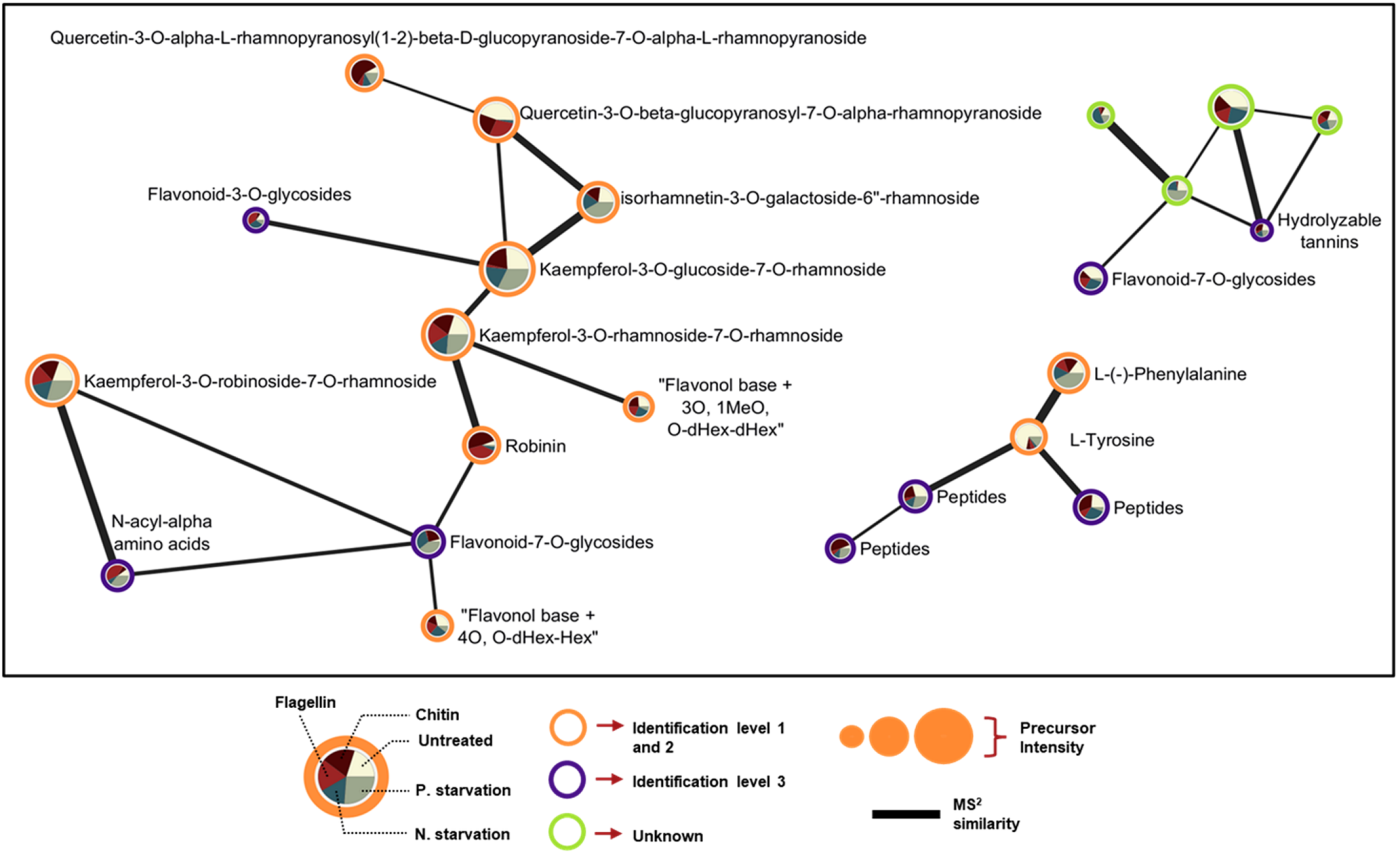
Selected regions of spectral similarity Molecular Networking of features detected in ESI^-^. Nodes (expressed as pie charts) represent specific metabolic features, and edges represent similarity based on the calculated Bonanza score. The thickness of edge lines is proportional to the calculated Bonanza score, while node size corresponds to the relative abundance of the metabolic feature in the extract. The segments in the pie charts represent the different treatments, with their sizes indicating the proportion of the average metabolite abundance for each treatment (n = 5) relative to the total abundance across all treatments. Outer ring color inside the nodes corresponds to the identification level.

Finally, we generated Molecular Networks of metabolic features with MS_2_ spectra after molecular formula and chemical ontology determination with MS-Finder. The connectivity of unknown metabolic features with metabolites identified with high confidence gave us an idea about the type of the unknown compound. Even metabolic features with the lowest identification level 3 retain useful information since they can be substrates to assayed transporters with a transportomics methodology. Verification of the chemical ontology could give information about what type of compounds can be associated with specific transporters or if genetically related transporters are able to interact with molecules belonging to the same class of compounds revealing information about the molecular recognition mechanism of the protein. Unidentified features connected with identified metabolites can be annotated with more confidence after determination of the chemical ontology. From the results we can see that 4 unidentified metabolic features connect with the flavonoids indicating similarity in their structures (**Figure 9**).

## 4 Discussion

We hereby report the establishment of an analytical framework for the generation, characterization and usage of plant extracts as complex substrate mixtures for oocyte-based transporter assays. We compared four different extraction protocols and identified a liquid-liquid extraction protocol as the best compromise between the chemical space extracted, extraction reproducibility, and oocyte permeation capability for transporter assays. To include stress-induced metabolites, we used liquid culture-grown Arabidopsis seedlings under artificially induced treatments mimicking four (a)biotic stresses (treatment with N- and P-starvation, flagellin 22 and chitin) and combined them into a final pooled extract containing treatment-specific metabolites. Metabolites included in the final extract were identified with a combination of pure standards, spectral database search and in-silico tools, enabling the identification of more than 200 compounds.

### 4.1 Metabolite content of extracts

In contrast with other untargeted metabolomics studies comparing different extraction solvent systems with respect to their capabilities in extracting a predefined pool of metabolites commonly found in the tissue of interest (Tulipani et al., 2013; Sitnikov et al., 2016; Schippers et al., 2023), we evaluated the four different extractions only with respect to the number of metabolic features detected in each without any information about the identity and diversity of the metabolites within different extractions. We believe that in the context of unbiased transporter characterization based on transportomics approaches, the extract used as substrate should not be discriminative towards any class of compounds but rather include as many metabolites from different compound classes as possible to maximize the chances of identifying new substrates. In accordance with their solvent compositions, the individual extraction protocols might include extraction-specific compounds, be more enriched, or completely lack specific metabolites. For example, the MeOH/H_2_O-based extraction protocol C, presented the highest number of features among the analyzed protocols, but it is likely that the increased number of features using this protocol does not directly reflect the metabolites captured, but possibly artifacts and redundant ions arising from degradation of biological polymers such as nucleic acids and polysaccharides (Duportet et al., 2012), metabolites generated during the extraction process (Sauerschnig et al., 2017), or adducts and in-source fragments generated during the ionization process (Mahieu and Patti, 2017). To discriminate between these features and features representing real plant metabolites other approaches such as the use of stable isotopes need to be employed (Doppler et al., 2016; Tsugawa et al., 2019). Since all four extraction protocols, susceptible to the aforementioned phenomena, presented comparable amounts of metabolites after filtration of the low-quality metabolic features, we concluded that the number of detected features is an important aspect of the selection of ideal extraction methodology but not be the only conclusive factor.

### 4.2 Inter- and intraday reproducibility of extracts

Another important aspect considered for the selection of the appropriate protocol is the inter-and intraday reproducibility. In accordance with previous studies (Sostare et al., 2018; Andresen et al., 2022; Yu and Huan, 2022), we used the median Relative Standard Deviation (intraday) and pooled Relative Standard Deviation (interday) as a measure of the total reproducibility from the different features among the same day and different days of measurements and did not evaluate reproducibility on the singular metabolite level. We consider reproducibility an important aspect of deciding on the optimal extraction protocol, since the metabolites detected among replicates of the same extract must be as similar as possible, to ensure uniformity of the metabolic space presented in different transporter proteins assayed with the same extract. On the same note, interday reproducibility reflects the performance of an extraction protocol on different days of measurement and can highlight the stability of an extract under prolonged storage conditions. Notably, besides the more complex experimental set-up, extraction protocol D presented a higher inter- and intraday reproducibility than the single-phase extraction protocol C contradicting previous studies (Zenati et al., 2023).

### 4.3 Lipophilic compounds and toxicity

Although extraction protocols A, B and C performed quite well on the above established criteria, their heavy load in pigments, lipids and lipophilic compounds could be a limiting factor for usage in oocyte-based screening assays. Proper resuspension of pellets generated from these extracts in Kulori buffer with small amounts of ethanol was difficult due to the distinct polarities of the resuspension solvent and the metabolites contained in the pellet. In consequence, this leads to high variability in the process even among technical replicates. More importantly, as highlighted through the toxicity assays, extract C required a greater extent of dilution compared to lipid-free protocol D, signifying that the presence of those compounds seems to increase the intrinsic toxicity of the extract as previously suspected (Maldifassi et al., 2016). We believe that the oocyte system as a heterologous expression host is not compatible with extract enriched in lipophilic molecules for transport assays since the latter can passively diffuse through the membrane and compromise the credibility of the results.

Based on the toxicity assay results, the major variable that affects oocyte permeation is the dilution of the extract used. Moreover, the extent of the measured permeation heavily depends on the signal-to-noise ratio of the analyte(s) selected, their initial concentration in the extract, as well as the biological batch-to-batch variability in oocytes, affecting their membrane susceptibility. We conclude that a liquid-liquid extraction such as the one used for protocol D displays a good compromise between the chemical space extracted, extraction reproducibility, and oocyte permeation capability.

### 4.4 Chitin diversity

The four treatments have successfully triggered metabolic changes in the plants since specific metabolic patterns can be associated with individual treatments. Surprisingly, from the four tested conditions, chitin-treated plant extracts present the least changes when compared to the untreated plant extracts. Previous studies have shown that chitin-treated plants exhibit distinct metabolic patterns such as changes in malondialdehyde levels, changes in the levels of primary metabolites involved in TCA cycle, carbon fixation and pyruvate metabolism as well as changes to individual secondary metabolites from various classes, none of which matched any of our treatment-related metabolites (Wan et al., 2008; Winkler et al., 2017; Zhang et al., 2017; Cabre et al., 2024).This might be attributed to the low concentration of active oligomers to trigger the metabolic response. It is known that commercial formulations of chitosan such as the one used in this study, contain a mixture of chitin oligomers with various polymerization degrees. Only a small proportion of these oligomers (pentamer to octamer) are responsible for eliciting plant immunity responses, thus an extra step of acidic hydrolysis before application might lead to the generation of more active oligomers and induce a stronger response (Winkler et al., 2017; Li et al., 2020).

### 4.5 ID of metabolites

The observed metabolites from the other three treatments (nitrogen starvation, phosphorus starvation and flagellin) were in accordance with published bibliography (Caldana et al., 2011; Krapp et al., 2011; Obata and Fernie, 2012; Yu et al., 2014; Frerigmann et al., 2016; Hiruma, 2019; Allwood et al., 2021; Godoy et al., 2021; Anzano et al., 2022; Offor et al., 2022; Xu and Fu, 2022; Xue et al., 2022; Cadena-Zamudio et al., 2023; Cabre et al., 2024). The final pooled extract composed of equal parts of the individual treatment extracts, presented a significantly increased number of features when compared to the untreated plant-derived extracts highlighting the success of treatments in increasing the metabolic space.

Combination of standard compounds, database search and in-silico tools alongside MS_2_ similarity networking can enhance metabolite identification and is a great strategy for characterization of plant extracts prior to utilization in oocyte uptake assays. Determination of the chemical ontology for compounds where no other information is available, can give a comprehensive overview of the metabolic diversity captured with the selected extraction, and provide knowledge of the chemical space presented on the oocytes and by extension to the transporters expressed on them. Combination of pure standards, spectra library matching, in -silico tools and spectral similarity molecular networking, is a powerful technique for metabolite identification that led to the assignment of more than 200 metabolite identities in our study.

## 5 Conclusion

In summary, we have created a protocol for generation of a plant extract with enhanced metabolite diversity that can be used as substrate mixture for oocyte-based transporter screening assays aimed at identification of transporter functionality. Our two-phase liquid-liquid extraction protocol presents reduced toxicity towards *Xenopus laevis* oocytes while maintaining the ability to capture a plethora of primary and secondary metabolites from different chemical classes.

## Data Availability

This data is available at the NIH Common Fund's National Metabolomics Data Repository (NMDR) website, the Metabolomics Workbench, https://www.metabolomicsworkbench.org, where it has been assigned Project ID PR002613. The data can be accessed directly via it's Project DOI: 10.21228/M8WK0T This work is supported by NIH grant, U2C- DK119886 (Sud et al., 2016).
