# Supplementary material for: Development of plant extracts as substrates for untargeted transporter substrate identification in Xenopus oocytes
Source: Front Plant Sci. 2025 Sep 17;16:1640426. doi: 10.3389/fpls.2025.1640426 (PMC12484206; doi:10.3389/fpls.2025.1640426)
Supplement: Supplementary file 1 [file DataSheet1.pdf]

Development of plant extracts as substrates for untargeted transporter substrate identification in *Xenopus* oocytes

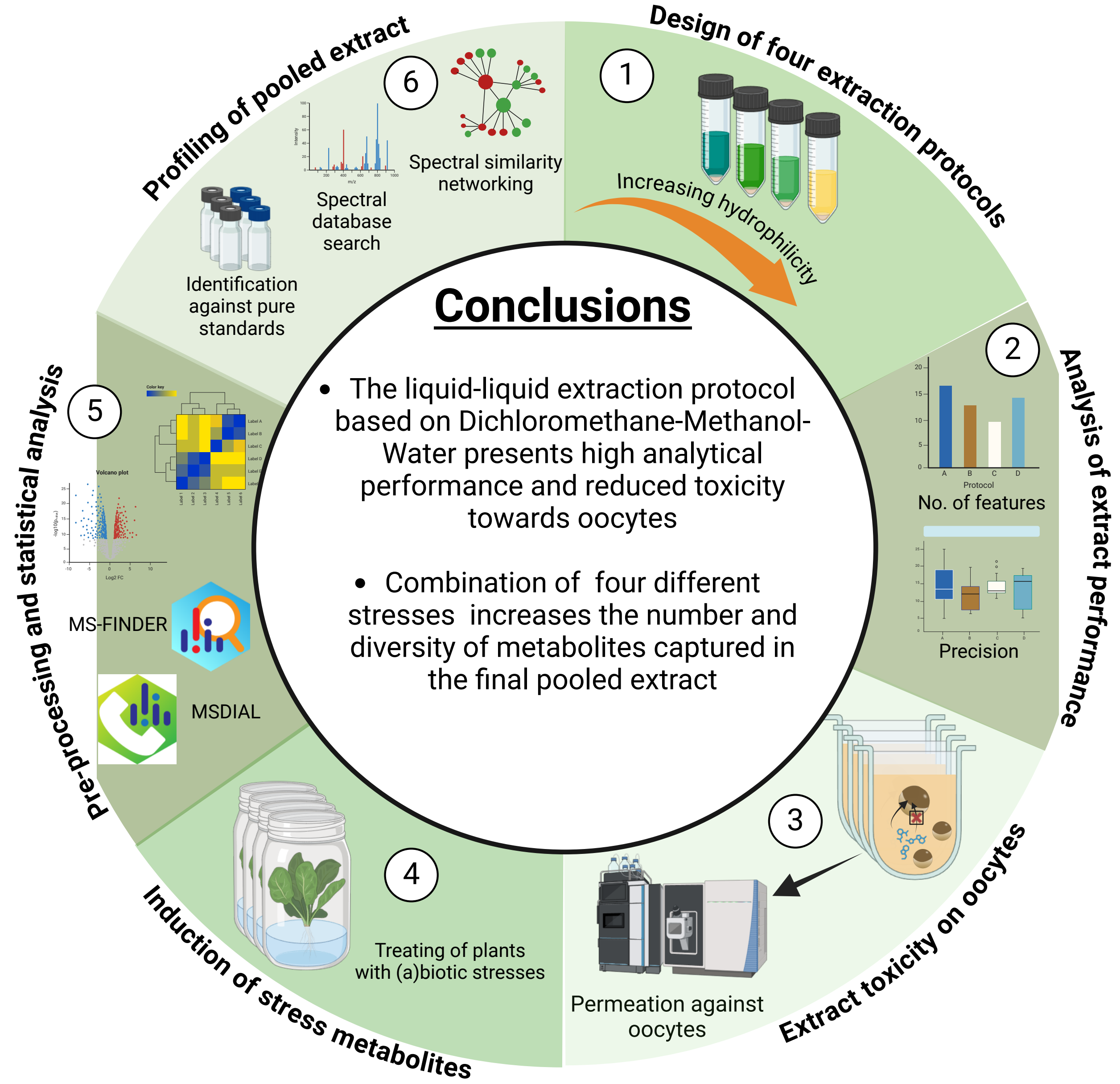

VILLUM FONDEN
