# Supplementary material for: Development of plant extracts as substrates for untargeted transporter substrate identification in Xenopus oocytes
Source: Front Plant Sci. 2025 Sep 17;16:1640426. doi: 10.3389/fpls.2025.1640426 (PMC12484206; doi:10.3389/fpls.2025.1640426)
Supplement: Supplementary file 2 [file DataSheet2.zip › Supplementary Material/README.-Supplementary.docx]

# ‘**Development of plant extracts as substrates for untargeted transporter substrate identification in *Xenopus* oocytes**

**This file contains information about the supplementary material of the present work.**

**File explanations**

- ‘**Supplementary File-1-Extraction-Protocol-Comparison’** contains the results from the individual multiple comparisons between different extraction protocols, calculations of inter- and intraday precision and statistical test results.
- ‘**Supplementary-File-2-Plant-treatment-Volcano-plots’** contains the results of the multiple comparisons between extracts originating from treated plants vs extracts originating from untreated plants.
- ‘**Supplementary-Table** **1**’ contains all metabolites identified against standards (Level 1) and spectral databases (Level 2)
- ‘**Supplementary-Table-2-Treatment-Comparison-ESI-** ’Contains all metabolic features detected in negative electrospray ionization as exported after pre-processing with MSDIAL
- ‘**Supplementary-Table-3-Treatment-Comparison-ESI+** ’Contains all metabolic features detected in positive electrospray ionization as exported after pre-processing with MSDIAL
- ‘**Supplementary Method 1’** contains all parameters for analysis with MSDIAL and MSFINDER
- **Supplementary-Figure 1** represents a schematic overview of the experimental work conducted in the paper.
- **Supplementary-Figure 2** represents the relevant physicochemical properties of NMOI3M selected as a permeation marker.
- **Supplementary-Figure 3** represents the comparison of extract C resuspensions with only Kulori buffer, 5% DMSO and 5% Ethanol in Kulori.
- **Supplementary-Figure 4** represents multiple comparisons between untreated plant extracts vs plant extracts from plants with individual treatments
