# Supplementary material for: Development of plant extracts as substrates for untargeted transporter substrate identification in Xenopus oocytes
Source: Front Plant Sci. 2025 Sep 17;16:1640426. doi: 10.3389/fpls.2025.1640426 (PMC12484206; doi:10.3389/fpls.2025.1640426)
Supplement: Supplementary file 2 [file DataSheet2.zip › Supplementary Material/Supplementary Figure 1.docx]

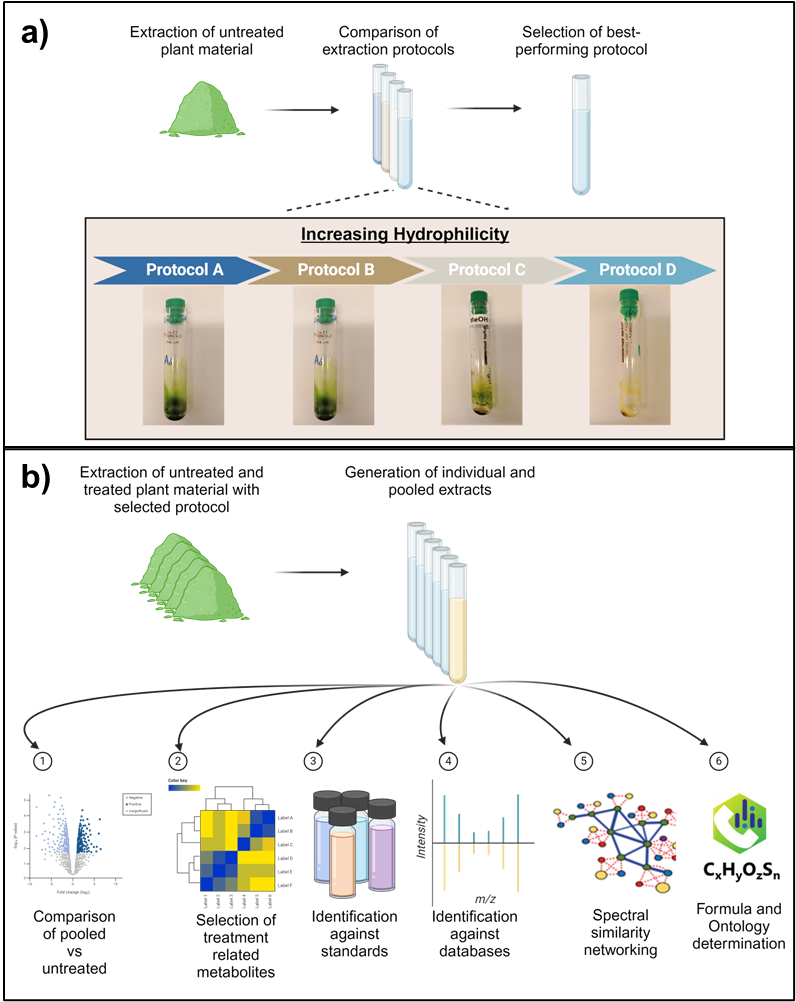


**Supplementary Figure 1**: Schematic overview of the experimental work conducted in the paper. a) Development and systematic evaluation of the four protocols for the selection of the ideal protocol for transportomics assays. b) Generation of treated and pooled extracts based on the selected protocol. Comparison of the pooled extract against the untreated plant extract and selection of treatment specific metabolites followed by a multilevel identification of metabolites with a combinatorial approach.
