# Supplementary material for: Development of plant extracts as substrates for untargeted transporter substrate identification in Xenopus oocytes
Source: Front Plant Sci. 2025 Sep 17;16:1640426. doi: 10.3389/fpls.2025.1640426 (PMC12484206; doi:10.3389/fpls.2025.1640426)
Supplement: Supplementary file 2 [file DataSheet2.zip › Supplementary Material/Supplementary Figure 2.docx]

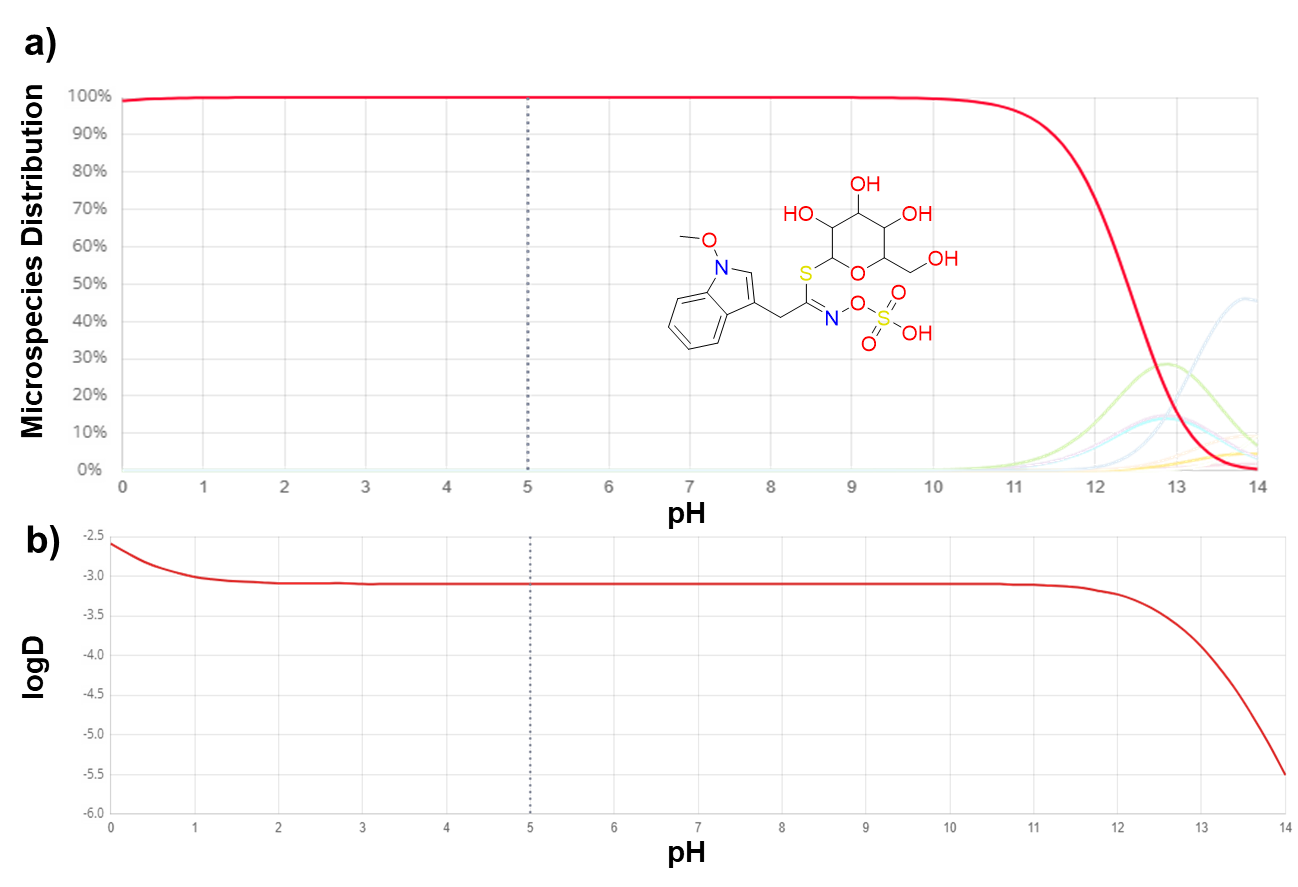


**Supplementary Figure 2:** Examination of physicochemical properties of NMOI3M. **a)** Microspecies distribution of NMOI3M in pH=5.**b)** Calculation of logD of NMOI3M in different pH. In pH=5, NMOI3M presents a negative logD value of -3.10 indicating high hydrophilic character. Calculations were conducted in MarvinSketch.
