# Supplementary material for: Development of plant extracts as substrates for untargeted transporter substrate identification in Xenopus oocytes
Source: Front Plant Sci. 2025 Sep 17;16:1640426. doi: 10.3389/fpls.2025.1640426 (PMC12484206; doi:10.3389/fpls.2025.1640426)
Supplement: Supplementary file 2 [file DataSheet2.zip › Supplementary Material/Supplementary Method 1.docx]

**Supplementary Method 1-Analysis with MS-DIAL and MS-Finder:** Parameters for analysis with MS-DIAL were as follows: MS1 tolerance:0.01 Da, MS2 tolerance:0.025; Retention time begin:0.5 minutes, retention time end: 10 minutes, MS1 range:50-1500, MS2 range:0-2000; Maximum charged number=2, Minimum peak height:100, Mass slice width:0.1 Da; Smoothing with Linear weighted moving average, Smoothing level: 3 scans, Minimum peak width: 5 scans, Annotation MS1 tolerance 0.01 Da and MS2:0.025 Da, Retention time tolerance:100 min, minimum reverse dot product score:0.8, Minimum peaks matched:4; Alignment retention time tolerance: 0.05 minutes, MS1 tolerance 0.015 Da.MS-Finder was used with spectral database search, formula predictions and structure elucidation with in-silico fragmenter as follows: For chemical formula calculations we used an Isotopic ratio tolerance of 20% and included C,H,O,N,P,S as possible elements. Top 5 structure candidates were kept after database search. Tree depth of in silico MS/Ms fragmenter: 2.
