# Supplementary material for: Development of plant extracts as substrates for untargeted transporter substrate identification in Xenopus oocytes
Source: Front Plant Sci. 2025 Sep 17;16:1640426. doi: 10.3389/fpls.2025.1640426 (PMC12484206; doi:10.3389/fpls.2025.1640426)
Supplement: Supplementary file 2 [file DataSheet2.zip › Supplementary Material/Supplementary Table 1.docx]

**Supplementary Table 1:** Identified metabolites in this study (levels1=pure standards and 2.1=Database search). Alignment ID corresponds to the Alignment ID plotted in Figure 9. For metabolites identified in both ionization modes both alignment IDs are being provided (e.g Arginine=448-neg/643-pos).Hex=hexoside,dHex=dihexoside, LPC=Lysophosphatidylcholine

| **Alignment ID** | **Metabolite name** | **Average Rt(min)** | **Average m/z** | **Adduct type** |
| --- | --- | --- | --- | --- |
|  |  |  |  |  |
| 540 | Spermidine | 0.737 | 146.17039 | [M+H]+ |
| 259 | 1-Pyrrolidinecarboximidamide | 0.803 | 114.1034 | [M+H]+ |
| 554 | L-Lysine | 0.813 | 147.10982 | [M+H]+ |
| 398 | (4-Aminobutyl)guanidine | 0.814 | 131.1299 | [M+H]+ |
| 626 | L-HISTIDINE | 0.878 | 156.07825 | [M+H]+ |
| 448-neg/643-pos | L-Arginine | 0.887 | 173.10396 | [M-H]- |
| 85 | L-Serine | 0.89 | 104.03422 | [M-H]- |
| 1298 | D-Ribose | 0.898 | 299.09442 | [M-H]- |
| 199-neg/408-pos | L-Asparagine | 0.902 | 131.04543 | [M-H]- |
| 831 | Arginine | 0.907 | 175.1196 | [M+H]+ |
| 108 | beta.-Cyano-L-alanine | 0.912 | 113.04218 | [M-H]- |
| 280-neg/551-pos | Glutamine | 0.913 | 145.06194 | [M-H]- |
| 940 | O-Phosphocholine | 0.933 | 184.07494 | [M]+ |
| 290 | N-Methyl-D-aspartic acid | 0.94 | 146.04568 | [M-H]- |
| 297 | L-Threonine | 0.94 | 120.06525 | [M+H]+ |
| 454 | N-Acetyl-L-aspartic acid | 0.951 | 174.0379 | [M-H]- |
| 652 | L-Citrulline | 0.954 | 159.07687 | [M+H-NH3]+ |
| 557 | Glutamic acid | 0.968 | 148.06206 | [M+H]+ |
| 404 | D-(+)-Ribonic acid .gamma.-lactone | 0.983 | 165.03825 | [M+OH]- |
| 1829 | Sucrose | 1.027 | 377.0842 | [M+Cl]- |
| 2103 | Glucoiberin | 1.039 | 422.02747 | [M-H]- |
| 2207 | (Methylsulfinyl)butyl glucosinolate | 1.041 | 436.0401 | [M-H]- |
| 209 | D-(+)-Malic acid | 1.184 | 133.0139 | [M-H]- |
| 127 | Maleic acid | 1.197 | 115.00671 | [M-H]- |
| 285 | Valine | 1.409 | 118.08687 | [M+H]+ |
| 382 | L-Pipecolic acid | 1.469 | 130.08704 | [M+H]+ |
| 245 | Ethyl imidazole-2-carboxylate | 1.471 | 113.03487 | [M+H-C2H4]+ |
| 537 | N-Acetyl-L-glutamine | 1.478 | 187.0732 | [M-H]- |
| 410-neg/763-pos | Uric acid | 1.538 | 167.01976 | [M-H]- |
| 576 | Methionine | 1.57 | 150.05853 | [M+H]+ |
| 1347-neg/2131-pos | Glutathione, reduced | 1.642 | 306.07535 | [M-H]- |
| 373-pos/181,182-neg | DL-Pyroglutamic acid | 1.893 | 130.05035 | [M+H]+ |
| 81 | 4-hydroxybutanoic acid | 1.937 | 103.03771 | [M-H]- |
| 547 | N-Acetyl-L-glutamic acid | 1.95 | 188.0565 | [M-H]- |
| 438 | DL-o-Tyrosine | 1.974 | 136.07686 | [M+H-CH2O2]+ |
| 926 | L-Tyrosine | 1.974 | 182.08205 | [M+H]+ |
| 2297 | Glucoalyssin | 1.997 | 450.06689 | [M-H]- |
| 141-pos/157-neg | Succinic acid | 2.003 | 117.01908 | [M-H]- |
| 1809 | Adenosine | 2.032 | 268.10608 | [M+H]+ |
| 295 | trans-Glutaconic acid | 2.052 | 147.02708 | [M-H]- |
| 406 | DL-Norleucine | 2.064 | 132.10274 | [M+H]+ |
| 2296 | Glucoalyssin | 2.071 | 450.05334 | [M-H]- |
| 405 | Isoleucine | 2.121 | 132.1026 | [M+H]+ |
| 318 | Picolinic acid | 2.215 | 124.04126 | [M+H]+ |
| 874 | 7-methanesulfinylheptan-1-amine | 2.27 | 178.12628 | [M+H]+ |
| 528 | 5-methanesulfinylpentanenitrile | 2.275 | 146.06046 | [M+H]+ |
| 2395 | (Methylsulfinyl)hexyl glucosinolate | 2.363 | 464.07294 | [M-H]- |
| 723 | 4-(Methylamino)-4-(3-pyridyl)butyric acid | 2.367 | 164.07115 | [M+H-CH5N]+ |
| 452 | 3-Aminobenzoic acid | 2.425 | 138.05084 | [M+H]+ |
| 493 | Ethyl trans-caffeate | 2.441 | 179.03792 | [M-H]- |
| 1526 | 2-amino-9-methanesulfinylnonanoic acid | 2.475 | 236.12746 | [M+H]+ |
| 869 | 5-Hydroxyindol-2-carboxylic acid | 2.479 | 178.05069 | [M+H]+ |
| 1567 | Indole + 1O, 1carboxy, O-Hex | 2.484 | 338.08789 | [M-H]- |
| 744-pos/398-neg | Phenylalanine | 2.508 | 166.08694 | [M+H]+ |
| 568 | Tropic acid | 2.51 | 149.06145 | [M+H-H2O]+ |
| 2381 | 4-Hydroxyglucobrassicin | 2.543 | 463.04904 | [M-H]- |
| 642 | N-Acetyl-L-proline | 2.544 | 158.08705 | [M+H]+ |
| 412 | Vanillic acid | 2.556 | 167.0349 | [M-H]- |
| 1502 | 4-(Hexopyranosyloxy)-3-methoxybenzoic acid | 2.556 | 329.08408 | [M-H]- |
| 1417 | Benzoic acid + 2O, O-Hex | 2.588 | 315.07565 | [M-H]- |
| 381 | L-Lysine | 2.592 | 130.0862 | [M+H]+ |
| 764-neg/1378-pos | Pantothenic acid | 2.639 | 218.10211 | [M-H]- |
| 1054 | 8-methanesulfinyloctan-1-amine | 2.708 | 192.14275 | [M+H]+ |
| 697 | 3-Aminocoumarin | 2.844 | 162.05907 | [M+H]+ |
| 2043 | 5'-S-Methyl-5'-thioadenosine | 2.869 | 298.09756 | [M+H]+ |
| 437 | Adenine | 2.87 | 136.06197 | [M+H]+ |
| 542 | (E)-3,4-(Methylenedioxy)cinnamaldehyde | 2.877 | 147.04436 | [M+H-CH2O]+ |
| 1518 | Di-p-coumaroylputrescine | 2.881 | 235.14468 | [M+H-C9H6O2]+ |
| 1649 | 2-amino-10-methanesulfinyldecanoic acid | 2.933 | 250.14874 | [M+H]+ |
| 2748 | (Methylsulfonyl)octyl glucosinolate | 2.965 | 508.09885 | [M-H]- |
| 654 | Tryptophan | 2.998 | 203.08168 | [M-H]- |
| 530 | 1H-Indole-4-carboxaldehyde | 3 | 146.06334 | [M+H]+ |
| 978 | DL-Indole-3-lactic acid | 3.001 | 188.07077 | [M+H-H2O]+ |
| 2077 | 4-Methylthiobutyl glucosinolate | 3.003 | 420.04697 | [M-H]- |
| 1197 | N-Acetyl-D-tryptophan | 3.007 | 205.09639 | [M+H-C2H2O]+ |
| 613 and 443 | 3,4-dihydroxybenzoic acid | 3.026 | 155.0332 | [M+H]+ |
| 1201 | Benzoic acid + 2O, O-Pen | 3.036 | 285.06116 | [M-H]- |
| 1877 | Coumaroyl agmatine (isomer 1) | 3.048 | 277.16623 | [M+H]+ |
| 393 | 4-Isopropyl-4'-methylchalcone | 3.067 | 131.04942 | [M+H-C10H14]+ |
| 712 | Coniferyl alcohol | 3.068 | 163.07603 | [M+H-H2O]+ |
| 499 | 4-((1E)-3-Hydroxyprop-1-en-1-yl)-2-methoxyphenyl 6-O-(4-carboxy-3-hydroxy-3-methylbutanoyl)-.beta.-D-glucopyranoside | 3.074 | 179.07018 | [M-H-C12H18O9]- |
| 1902 | Coniferyl alcohol + O-Hex | 3.074 | 387.12875 | [M+HCOO]- |
| 235 | Salicylic acid | 3.077 | 137.0229 | [M-H]- |
| 1603 | Coniferin | 3.078 | 341.12332 | [M-H]- |
| 405 | Caffeyl alcohol | 3.125 | 165.05621 | [M-H]- |
| 1490 | 3-(4-(.beta.-D-Glucopyranosyloxy)phenyl)propanoic acid | 3.125 | 327.10492 | [M-H]- |
| 2615 | (Methylsulfinyl)octyl glucosinolate | 3.169 | 492.10251 | [M-H]- |
| 1160 | 9-methanesulfinylnonanenitrile (isomer 1) | 3.178 | 202.12659 | [M+H]+ |
| 682 | 4-Methoxycinnamic acid | 3.202 | 161.0602 | [M+H-H2O]+ |
| 301 | Phthalic acid | 3.204 | 121.02708 | [M+H-CH2O2]+ |
| 463 | Salicylic acid | 3.204 | 139.0421 | [M+H]+ |
| 1296 | Benzoic acid + 1O, O-Hex | 3.207 | 299.07648 | [M-H]- |
| 238 | 4-Hydroxybenzoic acid | 3.21 | 137.02364 | [M-H]- |
| 1200 | Dihydrobenzoic acid pentose | 3.214 | 285.05786 | [M-H]- |
| 2273 | Indolylmethyl glucosinolate | 3.217 | 447.05374 | [M-H]- |
| 3581-neg/4407-pos | Flavonol base + 4O, O-dHex, O-Hex-dHex | 3.263 | 755.20587 | [M-H]- |
| 1878 | Coumaroyl agmatine (isomer 2) | 3.267 | 277.1666 | [M+H]+ |
| 4093 | Cyanidin 3,5-diglucoside | 3.271 | 611.15808 | [M+H]+ |
| 3211 | Flavonol base + 4O, O-dHex-Hex | 3.287 | 609.14661 | [M-H]- |
| 1968 | Magnolioside | 3.299 | 399.0918 | [M+CHO2]- |
| 1065 | 6-Hydroxy-7-methoxycoumarin | 3.3 | 193.0509 | [M+H]+ |
| 2559 | Scopolin | 3.3 | 355.10321 | [M+H]+ |
| 1471 | Coumaroyl Hexoside (isomer 1) | 3.341 | 325.08878 | [M-H]- |
| 671 | 2-Methylamino-1-(3,4-methylenedioxyphenyl)propan-1-one | 3.395 | 160.07568 | [M+H-CH4O2]+ |
| 3537-neg/4377-pos | Kaempferol-3-O-robinoside-7-O-rhamnoside | 3.407 | 739.20905 | [M-H]- |
| 4040 | Kaempferol-3-O-galactoside-7-O-rhamnoside | 3.414 | 595.16589 | [M+H]+ |
| 2127 | Feruloyl agmatine | 3.418 | 307.17709 | [M+H]+ |
| 488 | .alpha.-Methyl-DL-phenylalanine | 3.439 | 178.0892 | [M-H]- |
| 2292 | 3,5-dihydroxy-2-(4-hydroxyphenyl)-7-[3,4,5-trihydroxy-6-(hydroxymethyl)oxan-2-yl]oxy-2,3-dihydrochromen-4-one | 3.466 | 449.10672 | [M-H]- |
| 2162 | Sinapine | 3.476 | 310.16666 | [M]+ |
| 857 | 3-Hydroxy-4-methoxycinnamic acid | 3.489 | 177.05141 | [M+H-H2O]+ |
| 1152 | (R)-Pantetheine | 3.497 | 277.12692 | [M-H]- |
| 1472 | Coumaroyl Hexoside (isomer 2) | 3.498 | 325.09464 | [M-H]- |
| 3153 | Robinin | 3.506 | 593.13947 | [M-H-C6H10O4]- |
| 799 | Neohancoside D | 3.519 | 223.06085 | [M-H-C12H20O10]- |
| 1882 | Sinapoylhexoside | 3.52 | 385.11337 | [M-H]- |
| 1224 | 3-O-Caffeoyl-4-O-sinapoylquinic acid | 3.523 | 207.0659 | [M+H-C16H18O9]+ |
| 3579 | 2-(3,4-Dihydroxyphenyl)-5,7-dihydroxy-4-oxo-4H-chromen-3-yl 6-deoxy-.alpha.-L-mannopyranosyl-(1->3)-6-deoxy-.alpha.-L-mannopyranosyl-(1->6)hexopyranoside | 3.539 | 755.19617 | [M-H]- |
| 2198 | 5-Methylthio-n-pentyl glucosinolate | 3.564 | 434.06183 | [M-H]- |
| 2104 | Phenylethyl glucosinolate | 3.569 | 422.06021 | [M-H]- |
| 3210 | Quercetin-3-O-beta-glucopyranosyl-7-O-alpha-rhamnopyranoside | 3.593 | 609.14581 | [M-H]- |
| 3303 | Luteolin-8-C-glucoside | 3.594 | 449.10846 | [M+H]+ |
| 4094 | Quercetin-3-O-b-glucosyl-7-O-a-rhamnoside | 3.595 | 611.1615 | [M+H]+ |
| 3398 | 2-[4-[(3S,3aR,6S,6aR)-6-[3-methoxy-4-[3,4,5-trihydroxy-6-(hydroxymethyl)oxan-2-yl]oxyphenyl]-1,3,3a,4,6,6a-hexahydrofuro[3,4-c]furan-3-yl]-2-methoxyphenoxy]-6-(hydroxymethyl)oxane-3,4,5-triol | 3.602 | 681.23792 | [M-H]- |
| 2498 | 4-Methoxyglucobrassicin | 3.629 | 477.064 | [M-H]- |
| 861 | 8-Methylsulfinyloctyl isothiocyanate | 3.686 | 231.06903 | [M-2H]- |
| 4408 | Flavonol base + 3O, O-dHex, O-Hex-Hex | 3.687 | 757.22314 | [M+H]+ |
| 3580 | Cyanidin 3-(2G-glucosylrutinoside) | 3.691 | 755.20209 | [M-H]- |
| 4375 | Robinin | 3.784 | 741.21924 | [M+H]+ |
| 3114 | Glucoliquiritin | 3.786 | 579.17017 | [M-H]- |
| 1947 | Kaempferol 3-glucoside 7-rhamnoside | 3.826 | 287.05273 | [M+H-C12H20O9]+ |
| 3155-neg/4039-pos | Kaempferol-3-O-glucoside-7-O-rhamnoside | 3.833 | 593.15125 | [M-H]- |
| 1521 | Lidocaine standard | 3.864 | 235.18074 | [M+H]+ |
| 3301 | Quercetin 3,7-dirhamnoside | 3.868 | 449.10822 | [M+H-C6H10O4]+ |
| 440 | N-Acetyl-D-norleucine | 3.873 | 172.09683 | [M-H]- |
| 1066 | 7,8-Dihydroxy-4-methylcoumarin | 3.88 | 193.05544 | [M+H]+ |
| 693 | Indole-5-carboxylic acid | 3.888 | 162.05528 | [M+H]+ |
| 3260 | isorhamnetin-3-O-galactoside-6''-rhamnoside | 3.894 | 623.16083 | [M-H]- |
| 3385 | Flavone base + 3O, 1MeO, C-Hex | 3.897 | 463.12515 | [M+H]+ |
| 4136 | Flavonol base + 3O, 1MeO, O-Hex-dHex | 3.897 | 625.1756 | [M+H]+ |
| 3535-neg/4376-pos | Kaempferol-3-O-galactoside-6''-rhamnoside-3'''-rha | 3.899 | 739.20331 | [M-H]- |
| 3043 | 5-Hydroxy-2-(4-hydroxyphenyl)-4-oxo-3-(.beta.-D-xylopyranosyloxy)-4H-chromen-7-yl 6-deoxy-.alpha.-L-mannopyranoside | 3.929 | 563.14185 | [M-H]- |
| 202 | Iminodiacetic acid | 3.936 | 132.03046 | [M-H]- |
| 990 | Phenylacetylaspartic acid | 3.936 | 250.07138 | [M-H]- |
| 1986 | 4-Methylpentyl glucosinolate | 3.97 | 402.08884 | [M-H]- |
| 3191 | Kaempferol-3-O-rhamnoside | 4.112 | 433.11279 | [M+H]+ |
| 3100-neg/3982-pos | Kaempferol-3-O-rhamnoside-7-O-rhamnoside | 4.113 | 577.15533 | [M-H]- |
| 2499 | Indolylmethyl glucosinolate + 1MeO | 4.119 | 477.06448 | [M-H]- |
| 377 | Indole-3-acetamide | 4.12 | 130.06488 | [M+H-CH3NO]+ |
| 2165 | Apigenin-8-C-glucoside | 4.128 | 431.0928 | [M-H]- |
| 673 | Indoleacetaldehyde | 4.129 | 160.07669 | [M+H]+ |
| 2285 | Glucolesquerellin | 4.163 | 448.07617 | [M-H]- |
| 389 | trans-4-Coumaric acid | 4.181 | 163.03786 | [M-H]- |
| 3386 | 5,8-Dihydroxy-2-(4-hydroxyphenyl)-7-methoxy-4-oxo-4H-chromen-3-yl 6-deoxy-.alpha.-L-mannopyranoside | 4.182 | 463.1308 | [M+H]+ |
| 2384 | Quercetin-3-O-glucoside | 4.184 | 463.08524 | [M-H]- |
| 3200 | Flavonol base + 3O, 1MeO, O-dHex-dHex | 4.185 | 607.16309 | [M-H]- |
| 2841 | Clemastanin B | 4.212 | 521.20581 | [M-H]- |
| 671 | N-Acetyl-D-phenylalanine | 4.243 | 206.08308 | [M-H]- |
| 1914 | trans-piceid | 4.284 | 389.12222 | [M-H]- |
| 980 | 8-methanesulfinyloctanenitrile | 4.379 | 188.10979 | [M+H]+ |
| 800-neg/1431-pos | Sinapic acid | 4.391 | 223.0609 | [M-H]- |
| 3393 | Daphnin | 4.392 | 679.14423 | [M-H]- |
| 1572 | Sinapoyl malate | 4.393 | 339.07129 | [M-H]- |
| 1227 | 3,5-Dimethoxy-4-hydroxycinnamic acid | 4.395 | 207.06607 | [M+H-H2O]+ |
| 589 | trans-Ferulic acid | 4.41 | 193.05029 | [M-H]- |
| 388 | 3-Hydroxycinnamic acid | 4.416 | 163.0367 | [M-H]- |
| 3102 | Apigenin-7-O-neohesperidoside | 4.416 | 577.15942 | [M-H]- |
| 543 | p-Coumaric acid | 4.424 | 147.04517 | [M+H-H2O]+ |
| 883 | Methyl 4-hydroxycinnamate | 4.43 | 179.07474 | [M+H]+ |
| 3300 | Quercitrin | 4.503 | 449.10565 | [M+H]+ |
| 2275 | Quercetin-3-O-rhamnoside | 4.517 | 447.08627 | [M-H]- |
| 2501 | Petunidin-3-O-beta-glucoside | 4.537 | 477.09894 | [M-H]- |
| 2063 | Syringaresinol diglucoside | 4.538 | 417.15833 | [M-H]- |
| 947 | Indole-3-acetyl-L-alanine | 4.594 | 245.09586 | [M-H]- |
| 2191 | Naringenin-7-O-glucoside | 4.676 | 433.11929 | [M-H]- |
| 2072 | 4-Deoxyphloridzin | 4.709 | 419.12256 | [M-H]- |
| 2372 | (Methylsulfanyl)heptyl glucosinolate | 4.795 | 462.09262 | [M-H]- |
| 2516 | (Methylsulfinyl)heptyl glucosinolate | 4.801 | 478.08707 | [M-H]- |
| 3501 | Coumarin base + 1O, 1MeO, O-(C26H31O12) | 4.83 | 725.20105 | [M-H]- |
| 3148 | Disinapoyl Hexoside | 4.832 | 591.17169 | [M-H]- |
| 1048 | Methyl 6-hydroxy-1H-indole-3-carboxylate | 4.846 | 192.06599 | [M-C6H10O5+H]+ |
| 1948 | Cyanidin | 4.881 | 287.05466 | [M]+ |
| 2166 | Kaempferol-3-O-rhamnoside | 4.883 | 431.09717 | [M-H]- |
| 1226 | Scoparone | 5.066 | 207.06596 | [M+H]+ |
| 3147 | Acacetin-7-O-neohesperidoside | 5.068 | 591.17133 | [M-H]- |
| 2679 | Sinapoyl + C6H9O5 | 5.072 | 369.11972 | [M+H]+ |
| 1162 | 9-methanesulfinylnonanenitrile (isomer 2) | 5.116 | 202.1275 | [M+H]+ |
| 374 | Coniferyl aldehyde | 5.226 | 162.01962 | [M-H]- |
| 457 | 3-Indoleacetic acid | 5.227 | 174.05632 | [M-H]- |
| 1532 | I.S ampicillin | 5.234 | 333.09039 | [M-H]- |
| 236 | Shikimic acid | 5.323 | 137.02348 | [M-H]- |
| 2484 | (Methylsulfanyl)octyl glucosinolate | 5.384 | 476.1088 | [M-H]- |
| 843 | 1-methoxyindole-3-carbaldehyde | 5.418 | 176.07072 | [M+H]+ |
| 3192 | Kaempferol-7-O-rhamnoside | 5.447 | 433.11438 | [M+H]+ |
| 1229 | Ethyl (2E)-3-(4-hydroxy-3,5-dimethoxyphenyl)-2-propenoate | 5.503 | 207.06833 | [M+H]+ |
| 3146 | Disinapoyl Hexoside | 5.503 | 591.16949 | [M-H]- |
| 3959 | NCGbeta-D-Glucopyranoside, 2-methoxy-4-[tetrahydro-3a,6a-dihydroxy-4-(4-hydroxy-3-methoxyphenyl)-1H,3H-furo[3,4-c]furan-1-yl]phenyl | 5.51 | 575.17889 | [M+H]+ |
| 2256 | Nonyl glucosinolate | 6.01 | 444.13556 | [M-H]- |
| 1509 | 1-Isothiocyanato-8-(methylsulfinyl)-octane | 7.27 | 234.09999 | [2M+3H2O+2H]+ |
| 3698 | LPC 18:3 | 8.815 | 518.33051 | [M+H]+ |
| 2354 | Benzyldimethyltetradecylammonium cation | 9.453 | 332.32977 | [M]+ |
| 3712 | LPC 18:2 | 9.652 | 520.34259 | [M+H]+ |
| 1105 | 2-Hydroxypalmitic acid | 11.208 | 271.22433 | [M-H]- |
| 3156 | Quercetin 3,7-dirhamnoside | 12.81 | 593.15125 | [M-H]- |
