# Supplementary material for: Development of plant extracts as substrates for untargeted transporter substrate identification in Xenopus oocytes
Source: Front Plant Sci. 2025 Sep 17;16:1640426. doi: 10.3389/fpls.2025.1640426 (PMC12484206; doi:10.3389/fpls.2025.1640426)
Supplement: Supplementary file 2 [file DataSheet2.zip › Supplementary Material/Supplementary-Figure 3.docx]

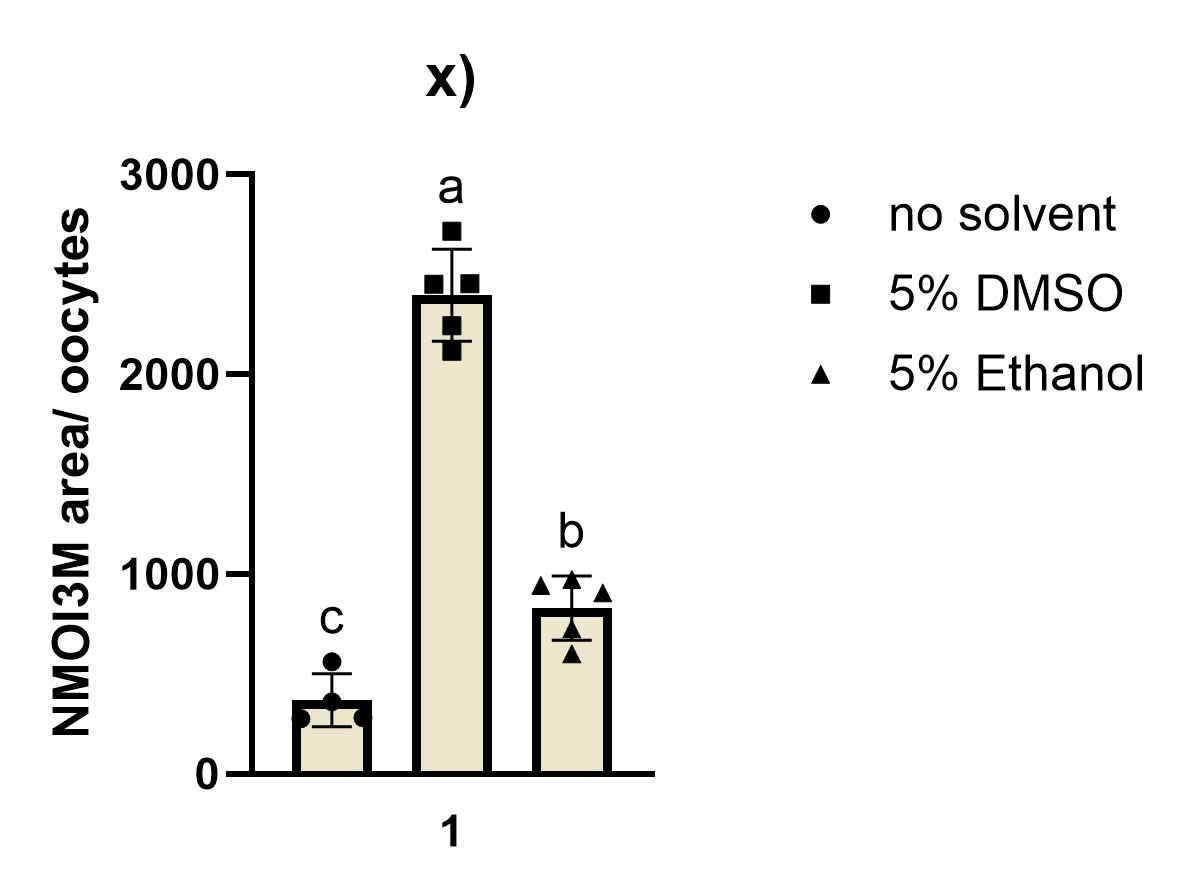


**Supplementary Figure 3:** Comparison of extract C resuspensions with only Kulori buffer, 5% DMSO and 5% Ethanol in Kulori. DMSO addition increases the permeating capabilities of extracts beyond Ethanol addition. Oocytes were assayed with medias derived from extract C and resuspended to 1 ml of volume with or without the addition of 5% v/v solvents. Samples of 5 oocytes were analysed for glucosinolate content. n=5. One-way ANOVA with Tukey’s multiple comparison correction. Different letters represent statistical differences.
