# Supplementary material for: Development of plant extracts as substrates for untargeted transporter substrate identification in Xenopus oocytes
Source: Front Plant Sci. 2025 Sep 17;16:1640426. doi: 10.3389/fpls.2025.1640426 (PMC12484206; doi:10.3389/fpls.2025.1640426)
Supplement: Supplementary file 2 [file DataSheet2.zip › Supplementary Material/Supplementary-Figure 4.docx]

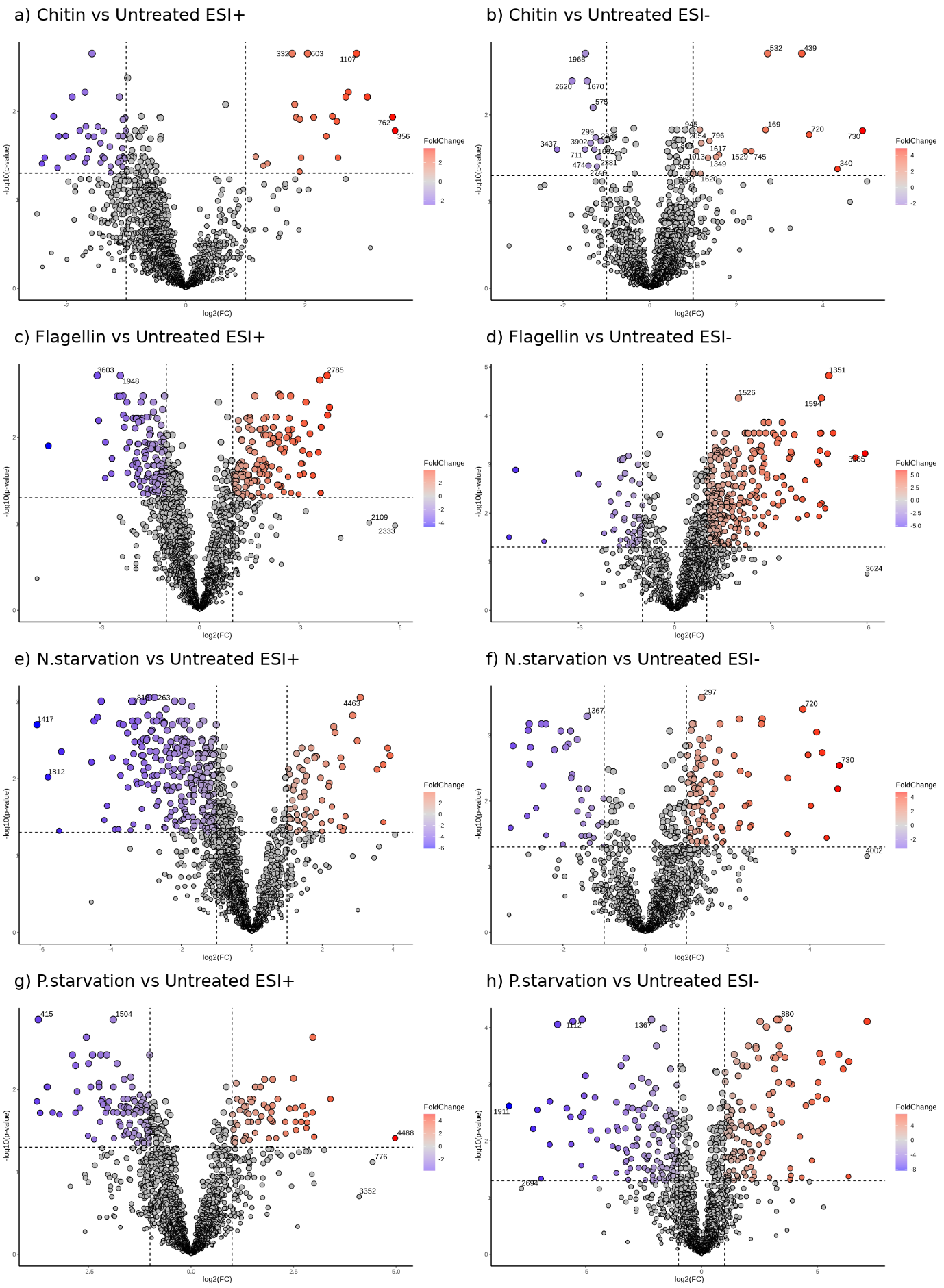


**Supplementary Figure 4:** Volcano plots representing multiple comparisons between untreated plant extracts vs plant extracts from plants with individual treatments in both ionization modes.
